# Remarkably selective and enantiodifferentiating sensing of histidine by a fluorescent homochiral Zn-MOF based on pyrene-tetralactic acid†
†Electronic supplementary information (ESI) available: General aspects, synthesis of starting materials and their spectral data, ^1^H and ^13^C NMR spectra of all the compounds, synthesis and X-ray crystal structure determination of **Zn-PLA**, fluorescence quenching titrations, determination of Stern–Volmer quenching constants and dye adsorption studies. CCDC 1429649. For ESI and crystallographic data in CIF or other electronic format see DOI: 10.1039/c5sc03839a


**DOI:** 10.1039/c5sc03839a

**Published:** 2016-01-07

**Authors:** Pujari Chandrasekhar, Arindam Mukhopadhyay, Govardhan Savitha, Jarugu Narasimha Moorthy

**Affiliations:** a Department of Chemistry , Indian Institute of Technology , Kanpur-208016 , India . Email: moorthy@iitk.ac.in

## Abstract

A highly luminescent and water-stable homochiral Zn-MOF, *i.e.*, **Zn-PLA**, has been developed based on a pyrene-tetralactic acid, which inherently features concave shapes for guest inclusion, to explore sensing of amino acids by fluorescence quenching; the solid-state fluorescence quantum yield of the MOF was found to be 46%. The fluorescence of an aqueous suspension of **Zn-PLA** was shown to be quenched specifically by histidine amongst all the other amino acids. Selective sensing of histidine is of prime importance due to its relevance in a variety of biological functions. The lack of quenching of fluorescence of **Zn-PLA** by all the amino acids other than histidine has been rationalized based on the exchange of the cationic dimethylammonium species in the MOF crystals with histidine that is protonated in water; the latter is envisaged to quench the fluorescence *via* charge transfer in the excited state. Furthermore, the homochiral crystals of **Zn-PLA** were found to permit enantiodiscrimination in the quenching by the d- and l-forms such that the ratio of enantioselectivity, *i.e.*, *K*_d_/*K*_l_, is 1.8, as determined by Stern–Volmer quenching plots. The highly selective as well as enantiodifferentiating sensing of amino acids by MOFs is unprecedented for any sensor type.

## Abstract 2

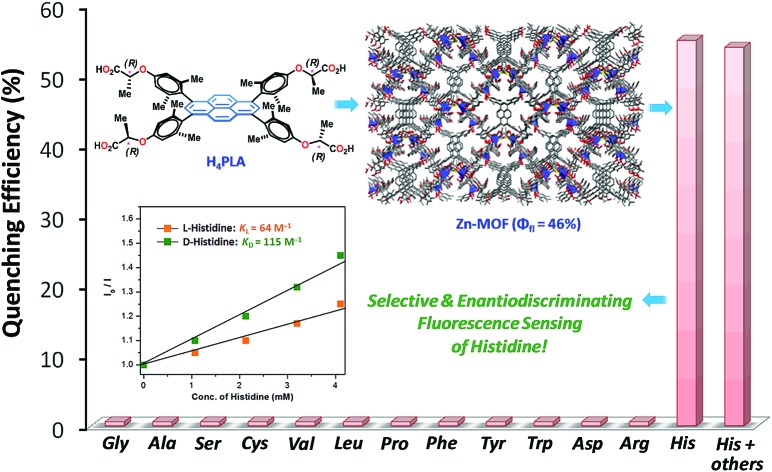
A highly luminescent and water-stable homochiral Zn-MOF, *i.e.*, **Zn-PLA**, developed based on pyrene-tetraacetic, selectively senses histidine amongst all other amino acids and also differentiates d and l isomers, as revealed by quenching of the fluorescence of its aqueous suspension.

## 1. Introduction

Amino acids are the essential ingredients of biological matter and are crucially involved in a variety of phenomena.1*a* Sensing of certain amino acids is important in the context of nutritional analysis1*b* and the diagnosis of diseases such as Alzheimers1*c* and pancreatitis.1*d* In particular, there has been a surge of interest in the sensing of histidine in biological fluids, as it is known to be associated with a variety of functions. In addition to being an essential amino acid for human growth,^2^ histidine is a neurotransmitter and a regulator of metal transmission in mammals.^3^ The human body uses histidine to manufacture histamine, which is responsible for a wide range of physiological processes.^4^ Furthermore, abnormal levels of histidine-rich proteins are known to manifest in a variety of ailments such as asthma and advanced liver cirrhosis.^5^ Amongst the generally employed techniques,^6^ which include colorimetric detection,6*a* capillary electrophoresis,6*b* and electrochemical methods,6*c* the fluorescence-based sensing of histidine is most attractive due to its rapid detection with high sensitivity. In fact, a variety of sensors based on fluorophore-crafted receptors, such as metal complexes,7*a* porphyrin,7*b* crown ethers,7*c* peptides,7*d* and polymers,7*e* have been reported for the selective detection of histidine. Although the design of sensors for histidine, one of the most basic amino acids, is simplified by the presence of an imidazole ring in the side chain; however, fluorescence-based sensing using some sensors has been noted to be interfered by other amino acids such as cysteine, tryptophan, tyrosine, lysine, and arginine.^8^ Significantly, chiral fluorescence-based sensors permit enantioselective sensing as well as an assay of the optically-enriched forms.^9^ The selective sensing of an amino acid and further enantiodiscrimination using fluorescence is challenging, and is heretofore unknown.

**Fig. 1:**
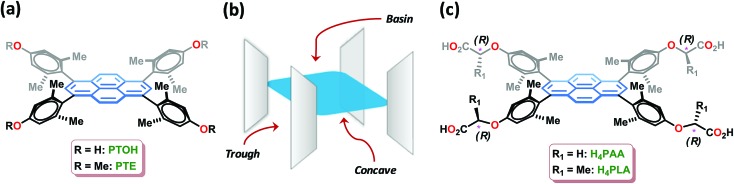
(a) Tetrarylpyrenes characterized by orthogonal planes and concave shapes. (b) A schematic of these molecular systems used to illustrate the three different domains, *viz.*, concave, trough and basin, used for guest inclusion. (c) The structures of the tetraacid linkers **H_4_PAA** and **H_4_PLA**.

Crystalline and porous metal–organic frameworks (MOFs) with luminescence properties, also termed LMOFs, have emerged as fascinating materials for sensing analytes bound in their pores.^10^ The luminescence in MOFs may arise as a consequence of one or more of the following: fluorescent organic linkers, luminescent metal ions such as lanthanide ions, a combination of both linkers and metal ions, fluorescent guest species that are bound by the MOFs, and scintillation.10*a* LMOFs have been extensively exploited during the last decade for sensing small molecules, volatile organic compounds (VOCs), explosive nitroaromatic compounds (NACs), odorants, transition metal ions, temperature, pH and biologically-relevant molecules.^10^ In particular, homochiral MOFs constructed from enantiopure chiral linkers have been demonstrated to be excellent for the enantioselective binding and separation of racemic mixtures.^11^ Although diverse MOFs have been developed for chiral resolution,^11^ porous homochiral LMOFs have been rarely explored, if at all, for the enantiodifferentiating fluorescence sensing of chiral analytes.^12^ Herein, we report the synthesis and application of homochiral fluorescent Zn-MOF, *i.e.*, **Zn-PLA** (Fig. 1), constructed from a rationally designed fluorescent pyrene-tetralactic acid linker that is endowed inherently with concave features,^13^ for the selective sensing as well as enantiodiscrimination of histidine in water.

## 2. Results and discussion

### 2.1. The design and synthesis of the chiral pyrene-lactic acid linker, **H_4_PLA**

In our comprehensive investigations entailing the rational design, synthesis, and demonstration of inclusion chemistry in the solid state, we have shown that molecular systems, such as **PTE** and **PTOH** (Fig. 1), which are characterized by orthogonal planes featuring three domains, namely, concave, trough and basin (Fig. 1), exhibit guest inclusion as a consequence of the creation of void spaces in their lattices.^13^ We have further shown that the metal-assisted assembly of such molecular systems leads to metal–organic materials that are necessarily porous.13*f* In our recent investigations, a fluorescent Mn-MOF-constructed from pyrene-acetic acid, *cf.***H_4_PAA** (Fig. 1), was shown to be porous and permitted the fluorescence sensing of solvent polarity and explosive nitroaromatics;13*f* the central pyrene core, which is protected by the orthogonally-oriented aryl rings at the four corners for quenching by the paramagnetic metal ions, is an excellent fluorophore. During these investigations, we discovered that the MOFs constructed with building blocks containing acetic acid moieties at the periphery, as found in **H_4_PAA**, are stable in water. This was indeed the basis for developing a chiral homologous pyrene-tetralactic acid, *i.e.*, **H_4_PLA**, to access homochiral fluorescent MOFs that can be used to explore the sensing of amino acids in water.

The tetraacid linker **H_4_PLA** was synthesized starting from its precursor tetraphenol **PTOH** (Fig. 1).13*f* A 4-fold Mitsunobu reaction using **PTOH** with (*S*)-(–)-methyl lactate in the presence of diisopropylazodicarboxylate (DIAD) furnished the chiral tetraester in 74% yield. Hydrolysis of the latter using K_2_CO_3_/MeOH led to the chiral tetralactic acid **H_4_PLA** in a near quantitative yield, *cf.* ESI.†

### 2.2. Synthesis and X-ray crystal structure determination of **Zn-PLA**

**Fig. 2:**
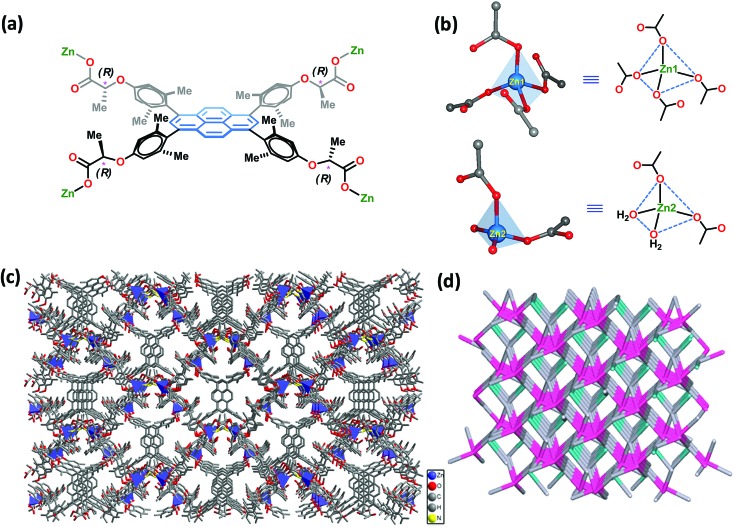
(a) The coordination modes of the carboxylate groups in **PLA** and (b) the coordination environment of Zn1 and Zn2 in **Zn-PLA**. (c) The crystal packing diagram of **Zn-PLA** along the *a*-axis. (d) The simplified topology of the **Zn-PLA** network.

The reaction of **H_4_PLA** with Zn(NO_3_)_2_·6H_2_O in *N*,*N*-dimethylformamide–H_2_O (4 : 1, v/v) solvent mixture in the presence of 45% aqueous HBF_4_ at 90 °C in a tightly-capped glass vial led to flaky crystals after 48 h. X-ray single crystal structure determination (deposition number: CCDC ; 1429649 †) revealed that the crystals belong to the monoclinic system with *I*2 space group, *cf.* ESI.† The asymmetric unit cell was found to contain 1.5 molecules of **PLA**, two Zn^2+^ metal ions, two coordinated water molecules and one dimethylammonium (DMA) cation. Thus, the molecular formula of the asymmetric repeating unit was [Zn_2_(**L**)_1.5_(H_2_O)_2_·Me_2_NH_2_]. The 1.5 molecules of **PLA** account for six units of negative charge, which is compensated by two Zn^2+^ ions and two DMA cations; unfortunately, one of the DMA cations could not be located due to the disorder in the large void volume. A careful analysis showed that one of these DMA cations is, indeed, stabilized inside the voids *via* strong hydrogen bonds with the oxygen atoms of the carboxylate groups of **PLA**, *cf.*, ESI.† The crystal structure analysis reveals that there exist two crystallographically independent Zn^2+^ ions, *i.e.*, Zn1 and Zn2, both of which are tetrahedral in geometry, *cf.*Fig. 2. Interestingly, Zn1 was found to be coordinated with four oxygen atoms from the four ligand carboxylate groups, whereas Zn2 was coordinated by four oxygen atoms from two ligand carboxylates and two water molecules. Thus, Zn1 behaves as a 4-connecting node, whereas Zn2 serves as a 2-connecting node. Fig. 2 shows the coordination modes of the carboxylate groups in **PLA**. The assembly of Zn^2+^ ions and the organic spacer **PLA** leads to the formation of a 3D anionic porous framework structure with channels that propagate down the *a*-axis (Fig. 2). The solvent accessible volume, which includes the missing DMA cation, in **Zn-PLA** MOF was calculated to be 35.5% using Mercury software (gridstep = 0.7 Å and probe radius = 1.2 Å). Interestingly, the 3D-framework of **Zn-PLA** corresponds to a new topology with a point symbol of (4.8^4^·10)_3_(4^3^·8^2^·10)_2_(8)_2_ and 2,4,4,4-*c* net with a stoichiometry of (2-*c*)_2_(4-*c*)_2_(4-*c*)_2_(4-*c*), as revealed by the TOPOS program. This was based on the consideration of two organic linkers as two 4-connecting units and two different metal ions as 2- and 4-connecting nodes. Fig. 2 shows the simplified topological network of the 4-nodal underlying net in a standard representation.

The synthesis of the Zn-MOF, *i.e.*, **Zn-PLA**, could be readily adapted to bulk scale synthesis; the MOF synthesized on a large scale was established to be identical to that of the X-ray determined single crystal by comparing the PXRD profile of pristine **Zn-PLA** with that simulated for the structure obtained using single crystal X-ray analysis, *vide infra*. Remarkably, the crystals of **Zn-PLA** were found to be highly stable when immersed in water for 1 day, as revealed by PXRD analysis, *vide infra*. The TGA of **Zn-PLA** was found to reveal solvent loss corresponding to *ca.* 20% up to 400 °C followed by thermal decomposition, *cf.* ESI.†

### 2.3. Fluorescence of chiral **Zn-PLA** and enantioselective fluorescence sensing of histidine

**Fig. 3:**
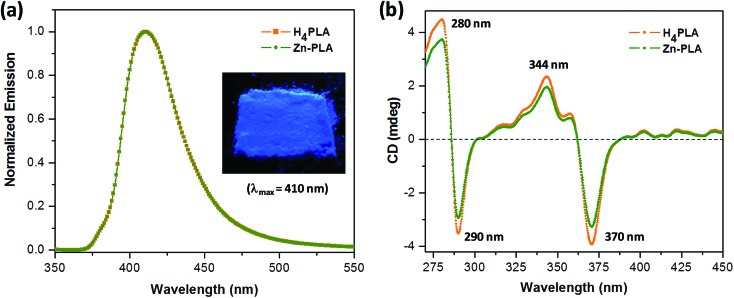
(a) The fluorescence emission spectra of **H_4_PLA** and **Zn-PLA** in the solid state (*λ*_ex_ = 350 nm). Notice that the emission spectra of **H_4_PLA** and **Zn-PLA** are identical. The inset shows the solid-state fluorescence image of the bulk sample of **Zn-PLA** when exposed to UV irradiation at 350 nm. (b) The solid-state CD spectra of **H_4_PLA** and **Zn-PLA**.

The crystals of **Zn-PLA** were found to display a brilliant blue emission upon exposure to UV light. Fig. 3 shows the solid-state fluorescence spectrum of **Zn-PLA** with an emission maximum at 410 nm for excitation at 350 nm. The solid-state fluorescence emission spectrum of the precursor organic linker **H_4_PLA** was found to be identical to that of **Zn-PLA**. This was not surprising given that the orthogonally-oriented aryl rings at the 4-corners of the pyrene moiety insulate the fluorescent pyrene core.13*f* The solid-state fluorescence quantum yields of **Zn-PLA** and **H_4_PLA** were determined by an integrating sphere setup to be 46% and 28%, respectively, for excitation at 350 nm. The enhancement in the fluorescence quantum yield of **Zn-PLA** when compared to that of the precursor organic linker **H_4_PLA** should be reconciled from differences in the environments around the pyrene core in the two structures; the conformational rigidity and highly ordered structure of the pyrene fluorophore within the framework presumably contribute to the observed increase in the fluorescence quantum yield of **Zn-PLA**.^14^ The latter is indeed a hallmark of MOFs, which deprive the fluorophoric linkers of their torsional freedom. In regard the chiroptical properties, the tetraacid linker **H_4_PLA** was found to display a moderate positive specific rotation of [*α*]25D = +15.8° in DMF. The solid-state CD spectra of both **H_4_PLA** and **Zn-PLA** show similar positive Cotton effects at *ca.* 280 and 344 nm and negative Cotton effects at *ca.* 290 and 370 nm, respectively, *cf.*Fig. 3. This substantiates the fact that the chirality of the organic linker is preserved in the Zn-MOF.

**Fig. 4:**
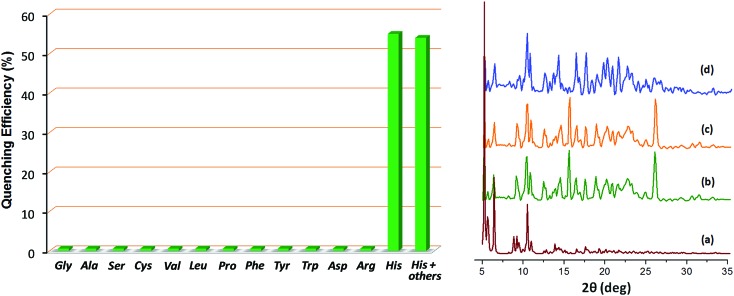
Left: The fluorescence quenching efficiencies of different l-amino acids upon addition to an aqueous suspension of **Zn-PLA**. The quenching efficiency was calculated for a 10 mM conc. of l-histidine and 100 mM conc. of all the other amino acids. Right: The PXRD profiles of **Zn-PLA**: (a) the simulated profile for the X-ray determined crystal structure, (b) pristine **Zn-PLA** MOF, (c) crystals immersed in distilled water, which were subsequently dried, and (d) crystals immersed in a 10 mM aqueous solution of l-histidine, which were subsequently dried.

The respectable fluorescence quantum yield, chirality, water stability and significant void volume of *ca.* 35% were the reasons to explore the utility of **Zn-PLA** to signal guest binding. To begin with, the fluorescence of the crystals of **Zn-PLA** suspended as a dispersion in water was examined in the presence of different types of natural l-amino acids, including small (glycine and alanine), nucleophilic (serine and cysteine), hydrophobic (valine, leucine and proline), aromatic (phenylalanine, tyrosine and tryptophan), acidic (aspartic acid) and basic (arginine and histidine) amino acids. Remarkably, a gradual decrease in the fluorescence intensity of the aqueous suspension of **Zn-PLA** was observed with an increasing concentration of l-histidine, whereas the fluorescence was virtually unaffected by all the other amino acids even at concentrations as high as 100 mM. For l-histidine employed at a 10 mM concentration, the quenching efficiency (*η*) was determined to be 55%; the quenching efficiency^15^ is defined as: 
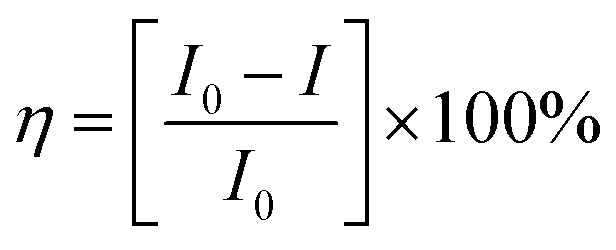
. Furthermore, the competition experiments showed that the fluorescence quenching efficiency of 10 mM l-histidine in the presence of all the other amino acids present at 100 mM concentration remained almost the same, *cf.*Fig. 4. Thus, the remarkable selectivity of the Zn-MOF to signal the binding of l-histidine over the other natural amino acids was clearly evident. The fact that the structural integrity of the crystals of **Zn-PLA** suspended in water and in the presence of histidine remains intact was established by PXRD analysis, Fig. 4.

**Fig. 5:**
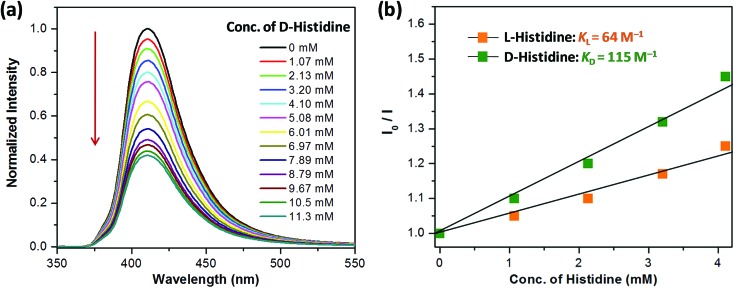
(a) Quenching of the fluorescence of **Zn-PLA** with an increasing concentration of d-(+)-histidine in water (*λ*_ex_ = 350 nm). (b) The Stern–Volmer quenching plots for d-(+)- and l-(–)-histidine.

Encouraged by the highly selective sensing of histidine by **Zn-PLA**, its ability to discriminate the enantiomers of histidine was examined. The fluorescence quenching titrations were performed with an increasing addition of d-(+)- and l-(–)-histidine to the dispersion of **Zn-PLA** in water. The fluorescence quenching titration of **Zn-PLA** with d-(+)-histidine is shown in Fig. 5. Indeed, the steady-state fluorescence quenching data for both d-(+) and l-(–)-histidine could be readily subjected to linear regression analysis of the Stern–Volmer plots; the Stern–Volmer equation is given by: 
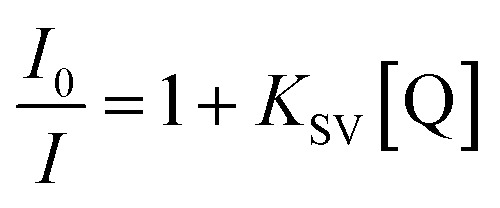
 Stern–Volmer constants, *i.e.*, *K*_d_ and *K*_l_ for d-(+)- and l-(–)-histidine, were determined to be 115 and 64 M^–1^, respectively. Evidently, the d and l isomers of histidine are differentiated by the Zn-MOF. The quenching of the fluorescence of **Zn-PLA** by d-(+)-histidine was thermodynamically more favorable than that found for l-(–)-histidine. It should be pointed out that an enantiodiscrimination of 51 M^–1^, *i.e.*, Δ*K* = (*K*_d_ – *K*_l_) and the enantioselectivity ratio of 1.8, *i.e.*, *K*_d_/*K*_l_, observed for **Zn-PLA** are akin to those achieved with ‘HELIXOL’,^16^ a well known helicene-based enantioselective fluorescent sensor used for sensing amines and amino alcohols.

### 2.4. The mechanism of quenching of the fluorescence of **Zn-PLA** by histidine

The emission of **Zn-PLA** essentially arises from the pyrene core, as evident from the superimposed solid-state fluorescence spectra of **H_4_PLA** and **Zn-PLA** shown in Fig. 3. The fact that the fluorescence of aromatic systems, such as anthracene and pyrene, is quenched by thiols, amines, and aromatic N-heterocycles such as imidazole *via* the photoinduced electron transfer (PET) mechanism has been well established.^17^ Given that the amino acids exist in their zwitter ionic forms, it was anticipated that amino acids, such as cysteine, histidine, tryptophan and tyrosine, would quench the fluorescence of **Zn-PLA**. Thus, selective sensing of the amino acids that contain thiol, imidazole, indole and hydroxyphenyl groups was expected. Indeed, we established from independent quenching studies on a model system, *i.e.*, pyrene-tetraether **PTE**, which is devoid of lactic acid moieties on the periphery, that the fluorescence of the latter was quenched by cysteine, histidine, tryptophan and tyrosine with diffusion limited rate constants, *cf.* ESI.† 18 The quenching rate constants determined for **PTE** were as follows: *k*_q_(Cys) = 3.7 × 10^9^ M^–1^ s^–1^; *k*_q_(His) = 3.9 × 10^9^ M^–1^ s^–1^; *k*_q_(Trp) = 5.1 × 10^9^ M^–1^ s^–1^; and *k*_q_(Tyr) = 6.9 × 10^9^ M^–1^ s^–1^. These rate constants were determined based on the steady-state Stern–Volmer quenching constants obtained for quenching of the fluorescence of **PTE** in DMSO and its singlet lifetime; the latter was determined to be 18.3 ns using nanosecond time-resolved studies, *cf.* ESI.† In light of these results, the observation that the fluorescence of **Zn-PLA** is not quenched by any of the 20 amino acids other than histidine is remarkable. Most surprising is the fact that the fluorescence of Zn-MOF is not influenced by amino acids such as cysteine, tryptophan and tyrosine. This underscores the unique environment offered by the MOF *vis-a-vis***PTE**; the fluorescence in both cases emanates from the central pyrene core.

The origin of such a remarkable selectively in the quenching by histidine should be reconciled from the attributes of histidine in comparison to other amino acids. The lack of quenching by all the amino acids with the exception of histidine suggests that they either cannot quench or those that are capable of quenching cannot establish the required proximity to the pyrene central core that is protected. The fact that the latter is indeed the case was established based on quenching studies with a model compound, *i.e.*, **PTE**, whose fluorescence was quenched by other amino acids as well. Furthermore, the selectivity also establishes the fact that the quenching observed with histidine cannot be a surface phenomenon, for the quenching should otherwise be observed for amino acids such as tryptophan and tyrosine.

Histidine is one of the three basic amino acids, which exist as cationic species in an aqueous medium.^19^ We suspected that the anionic **Zn-PLA** with DMA species as the counter cations presumably undergoes cation exchange with these amino acids when suspended in their aqueous solutions. Independently, the ability of **Zn-PLA** crystals to undergo post-synthetic exchange (PSE)^20^ of the DMA cations was investigated by suspending the MOF crystals in a solution of a cationic dye, namely, methylene blue. Indeed, the crystals of **Zn-PLA** were found to adsorb the cationic dye, *i.e.*, methylene blue, selectively without touching the anionic bromophenol blue and neutral nile red dye, see Fig. S11, ESI;† the blue color of the dye permits dye exchange to be observed by the naked eye *via* the blue coloration of the crystals. This suggests that **Zn-PLA** may undergo post-synthetic cation exchange with all the three basic amino acids that become cationic in the aqueous medium, leading to transport of these amino acids into the interiors of the crystals of **Zn-PLA**. Evidently, this should not be feasible for all the other amino acids. Why is it then that the fluorescence of **Zn-PLA** was only influenced by histidine and not by the other two basic amino acids, *i.e.*, arginine and lysine?

Histidine contains a 5-membered protonated imidazole aromatic ring, whereas arginine and lysine contain guanidine and amino groups that are protonated. The transport of histidine into the void spaces^21^ of **Zn-PLA** may lead to location of the protonated and electron-deficient imidazolium ring in the proximity of the pyrene core in **Zn-PLA** by which the fluorescence of the latter may be quenched *via* charge transfer in the excited state.13*f* Similar charge-transfer quenching will not apply to the protonated arginine and lysine, although the latter may also serve to exchange the DMA cations. Indeed, a closer inspection of the crystal structure reveals that the void spaces exist near the central pyrene core, *cf.* ESI.† Furthermore, the quenching efficiency was evidently dictated by orientational preferences, as is borne out from the observed enantiodiscrimination. The d-amino acid quenches more efficiently than the l-isomer, reflecting thereby a small energy difference in the diastereomeric geometries of the two isomers within the chiral MOF.

## 3. Conclusions

A chiral organic linker **H_4_PLA** with inherent concave shapes for guest inclusion was rationally designed to access porous MOFs using metal-assisted self assembly. Treatment of **H_4_PLA** with Zn(NO_3_)_2_ led to porous and luminescent crystals of **Zn-PLA**, for which the solid-state fluorescence quantum yield is 46%. The fluorescence of aqueous dispersion of **Zn-PLA** was quenched specifically by histidine amongst all the other amino acids, leading to its selective sensing. Given that the fluorescence of the model organic compound that is devoid of carboxyl groups is quenched by amino acids, such as cysteine, histidine, tryptophan and tyrosine with diffusion limited rates, the selective quenching of fluorescence of **Zn-PLA** by histidine is remarkable. The reason for the latter has been rationalized based on exchange of the cationic DMA species in the MOF crystals by histidine, which is protonated in water. The cationic imidazolium ring of histidine has been proposed to be involved in charge-transfer interactions with the protected pyrene fluorophore in its excited state for the observed quenching. The chirality existing in the MOF was imparted by the lactic acid moieties and allowed the enantiodiscrimination of the d and l forms of histidine. The ratio of enantioselectivity, *i.e.*, *K*_d_/*K*_l_, was determined to be 1.8 from the Stern–Volmer quenching plots. These results constitute the first examples of the highly selective and enantiodifferentiating sensing of an amino acid, namely, histidine, using MOFs in water.

## Supplementary Material

Supplementary information

Crystal structure data
