# Supplementary material for: Remarkably selective and enantiodifferentiating sensing of histidine by a fluorescent homochiral Zn-MOF based on pyrene-tetralactic acid
Source: Chem Sci. 2016 Jan 7;7(5):3085–91. doi: 10.1039/c5sc03839a (PMC6005210; doi:10.1039/c5sc03839a)
Supplement: Supplementary file 1 [file SC-007-C5SC03839A-s001.pdf]

Electronic Supplementary Material (ESI) for Chemical Science.
This journal is © The Royal Society of Chemistry 2016

ELECTRONIC SUPPLEMENTARY INFORMATION

Remarkably Selective and Enantiodifferentiating Sensing of Histidine by a Fluorescent Homochiral Zn-MOF Based on Pyrene-tetralactic Acid

Pujari Chandrasekhar,[§] Arindam Mukhopadhyay,[§] Govardhan Savitha,[§] and Jarugu Narasimha Moorthy[§,*]

[§] *Department of Chemistry, Indian Institute of Technology, Kanpur-208016, INDIA*

E-mail: moorthy@iitk.ac.in

CONTENTS

1.	General aspects	S2
2.	Synthesis of **H_4PLA**	S3
3.	^{1}H and ^{13}C NMR spectra	S5
4.	Solvothermal synthesis of **Zn-PLA**	S7
5.	X-ray crystallography	S7
6.	Characterization of **Zn-PLA**	S12
7.	Steady-state fluorescence spectroscopy	S13
8.	Fluorescence quenching titration experiments with **Zn-PLA**	S13
9.	Fluorescence quenching experiments with **PTE**	S14
10.	Postsynthetic cation-dye exchange studies	S17
11.	Tables of important crystallographic parameters of **Zn-PLA**	S18

General Aspects. ^{1}H NMR spectra were recorded with a JEOL-Lambda (500 MHz) spectrometer. ^{13}C NMR spectra were recorded with a 125 MHz NMR spectrometer with complete proton decoupling. IR spectra were recorded using a Bruker Vector 22 FT-IR spectrophotometer. Mass spectral analyses were carried out with Waters ESI-^{Q}TOF Instrument. Powder X-ray diffractograms (PXRDs) were recorded on a Bruker D8 Advance series 2 powder X-ray diffractometer. Thermogravimetric analyses (N_2 atmosphere, heating rate of 10 °C/min) were carried out with a Mettler Toledo TGA apparatus. The specific rotation of **H_4PLA** was determined with an AUTOPOL® IV automatic polarimeter (Rudolph Research Analytical, NJ, USA) using a standard 6 mL–cell (path length = 10 cm) at the wavelength of sodium-D (λ = 589 nm) line at 25 ^{o}C. The solid-state CD spectra were recorded using a JASCO J-815 CD spectrometer (with an accuracy of ± 1 nm) flushed with dry nitrogen at 25 ^{o}C. The melting points were determined with a JSGW melting point apparatus. The microscopic image of the crystals was recorded with a Nikon digital microscope. All the solvents were freshly distilled prior to use. (*S*)-(–)-Methyl lactate and diisopropylazodicarboxylate were obtained from Sigma-Aldrich, India. *N*,*N*-dimethylformamide and all the metal nitrates were obtained from Merck, India. HPLC grade water was obtained from Fisher Scientific, India. D-(+)- and L-(–)-histidine were obtained from Alfa Aesar, India.

Synthesis of the Chiral Tetracarboxylic Acid Linker H_4PLA

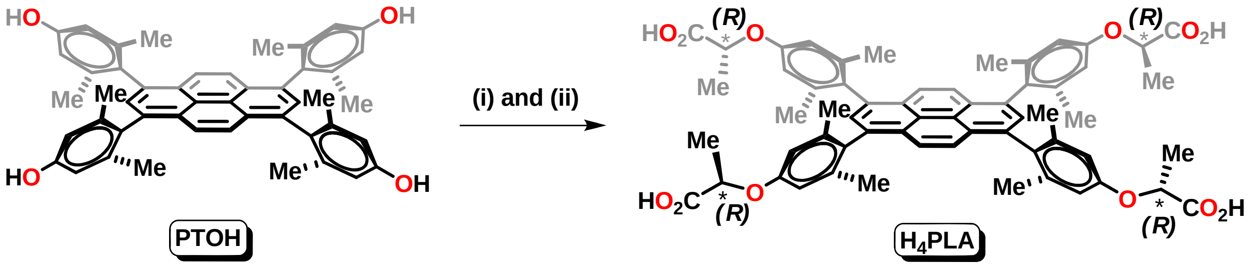

(i) (*S*)-Methyl lactate, PPh_3, DIAD, THF, rt, 2h (74%); (ii) K_2CO_3/ MeOH, 40 °C, 6h (94%)

1,3,6,8-Tetrakis(4-(α-carbomethoxy–(*R*)-α-methyl)methoxy-2,6-dimethylphenyl)pyrene.

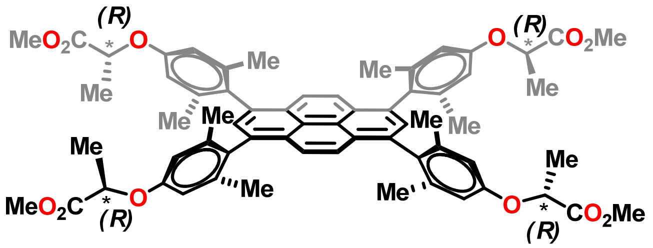

A 5 mL single neck round bottomed flask was charged with 1,3,6,8-tetrakis(2,6-dimethyl-4-hydroxyphenyl)pyrene **PTOH** (0.250 g, 0.366 mmol), triphenylphosphine (0.770 g, 3 mmol), (*S*)-(–)-methyl lactate (0.3 mL, 3 mmol) and dry THF (0.5 mL). The reaction mixture was sonicated for 10 min until a clear solution appeared. Subsequently, diisopropylazodicarboxylate (0.59 mL, 3 mmol) was added dropwise to the reaction mixture and it was allowed to sonicate for 2h. After completion of the reaction, the organic content was extracted with ethyl acetate and washed with brine. The organic layer was dried over anhyd Na_2SO_4, filtered and evaporated in vacuo. The crude material was subjected to silica gel column chromatography using 25% ethyl acetate/ hexane to afford 1,3,6,8-tetrakis(4-(α-carbomethoxy–(*R*)-α-methyl)methoxy-2,6-dimethylphenyl)pyrene as white solid; yield 76%; mp 272-276 °C; IR (KBr) cm^{-1} 2987, 2918, 2850, 1760, 1607, 1475, 1376, 1313, 1154; ^1H NMR (400 MHz, $CDCl_3$) δ 1.66 (d, J = 6.8 Hz,

12H), 1.92 (s, 24H), 3.80 (s, 12H), 4.84 (q, J = 6.8 Hz, 4H), 6.71 (s, 8H), 7.52 (s, 4H), 7.55 (s, 2H); ^{13}C NMR (100 MHz, $CDCl_3$) δ 18.7, 21.0, 52.3, 72.5, 113.7, 113.8, 124.8, 125.8, 128.6, 129.4, 133.5, 135.8, 138.4, 156.6, 172.9; HRMS m/z calcd for $C_{64}H_{66}O_{12}Na$ 1049.4452, found 1049.4490 $[M+Na]^+$.

1,3,6,8 -Tetrakis(4-(α-carboxy–(*R*)-α-methyl)methoxy-2,6-dimethylphenyl)pyrene, H_4PLA. A mixture of the above tetraester (0.660 g, 0.6 mmol), K_2CO_3 (0.835 g, 6 mmol) and methanol (25 mL) was stirred at 40 ^{o}C for 6 h. Subsequently, the solvent was evaporated under reduced pressure and the resultant solid residue was dissolved in 20 mL of distilled water. This solution was then washed twice with EtOAc to remove organic impurities and the aqueous phase was neutralized with 20% aq. HCl followed by extraction with EtOAc. The organic layer was then dried over anhyd Na_2SO_4 and concentrated in vacuo. The crude product was triturated with 20% EtOAc/pet. ether and the resulting precipitate was filtered to obtain **H_4PLA** as a white solid; yield 94%; mp > 300 °C; IR (KBr) cm^{-1} 3444, 2981, 2466, 1668, 1606, 1474, 1378, 1314, 1148; ^{1}H NMR (500 MHz, DMSO-d_6) δ 1.52 (d, J = 6.7 Hz, 12H), 1.85 (s, 24H), 4.88 (q, J = 6.7 Hz, 4H), 6.76 (s, 8H); 7.45 (s, 4H); 7.48 (s, 2H); ^{13}C NMR (125 MHz, DMSO-d_6) δ 18.5, 20.8, 71.5, 113.6, 114.1, 124.6, 125.3, 128.1, 129.8, 132.1, 135.9, 137.7, 156.8, 173.4; HRMS m/z calcd for $C_{60}H_{62}O_{12}N_1$ 988.4272, found 988.4278 $[M+NH_4]^+$.

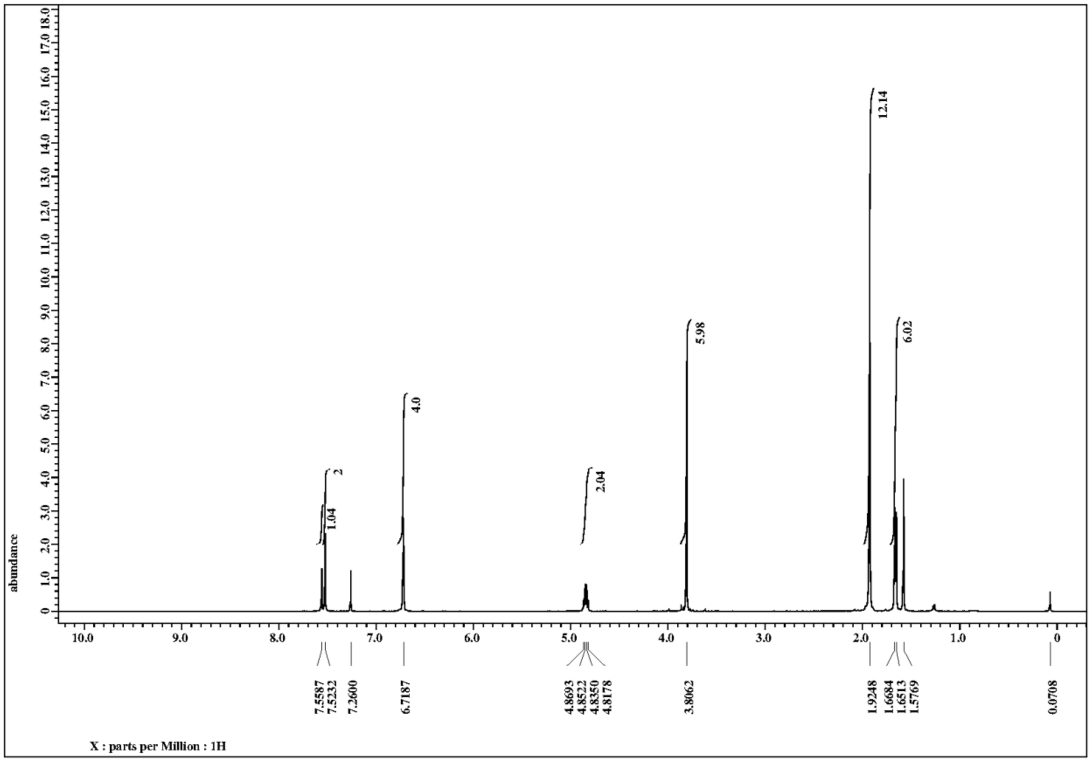

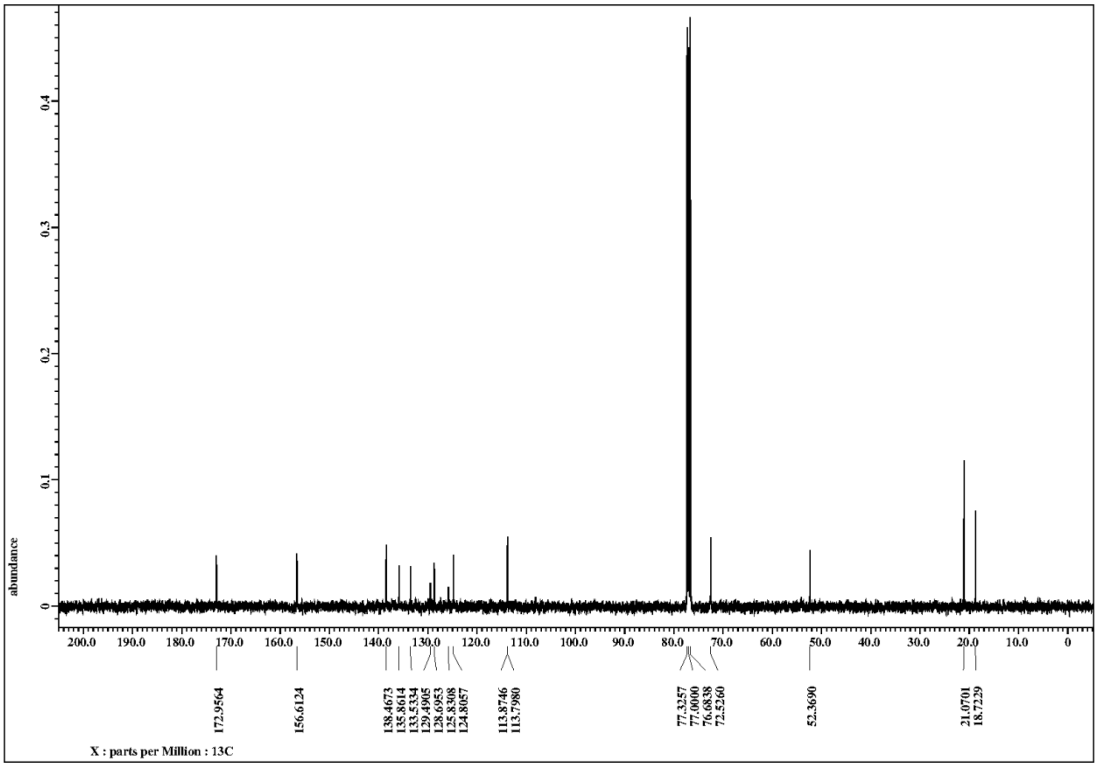

Figure S1. ^{1}H (400 MHz, $CDCl_3$) and ^{13}C NMR (100 MHz, $CDCl_3$) spectra of 1,3,6,8-tetrakis(4-(α-carbomethoxy–(R)-α-methyl)methoxy-2,6-dimethylphenyl)pyrene.

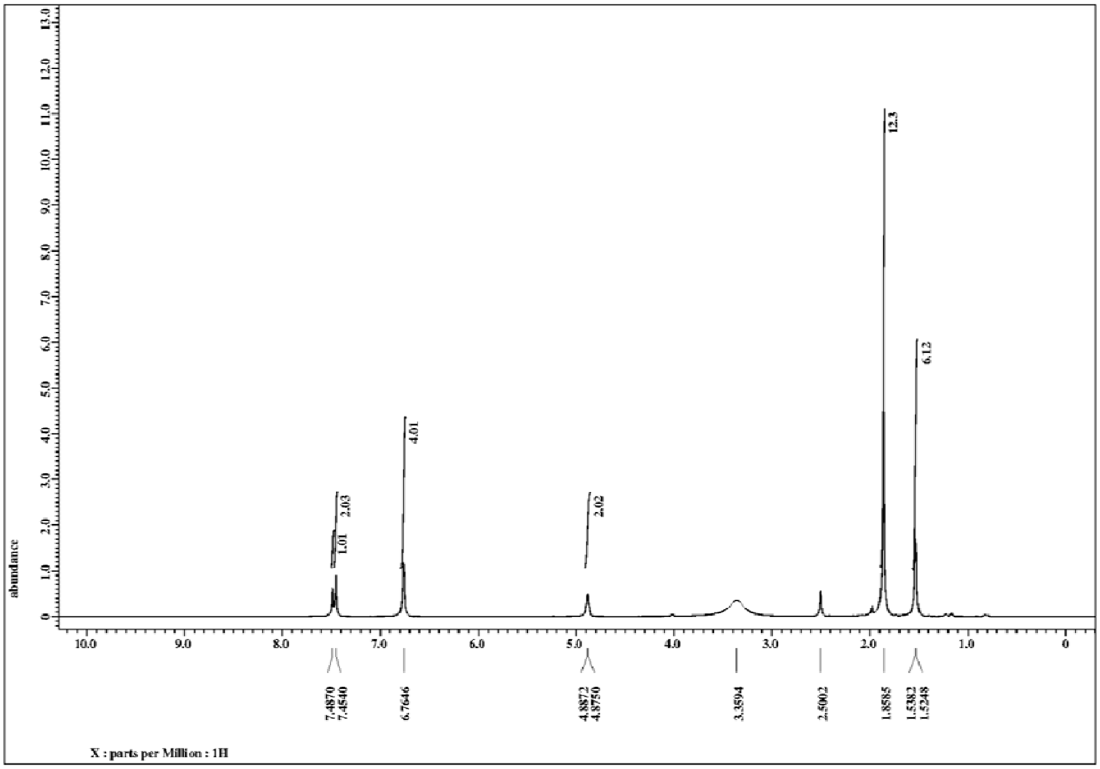

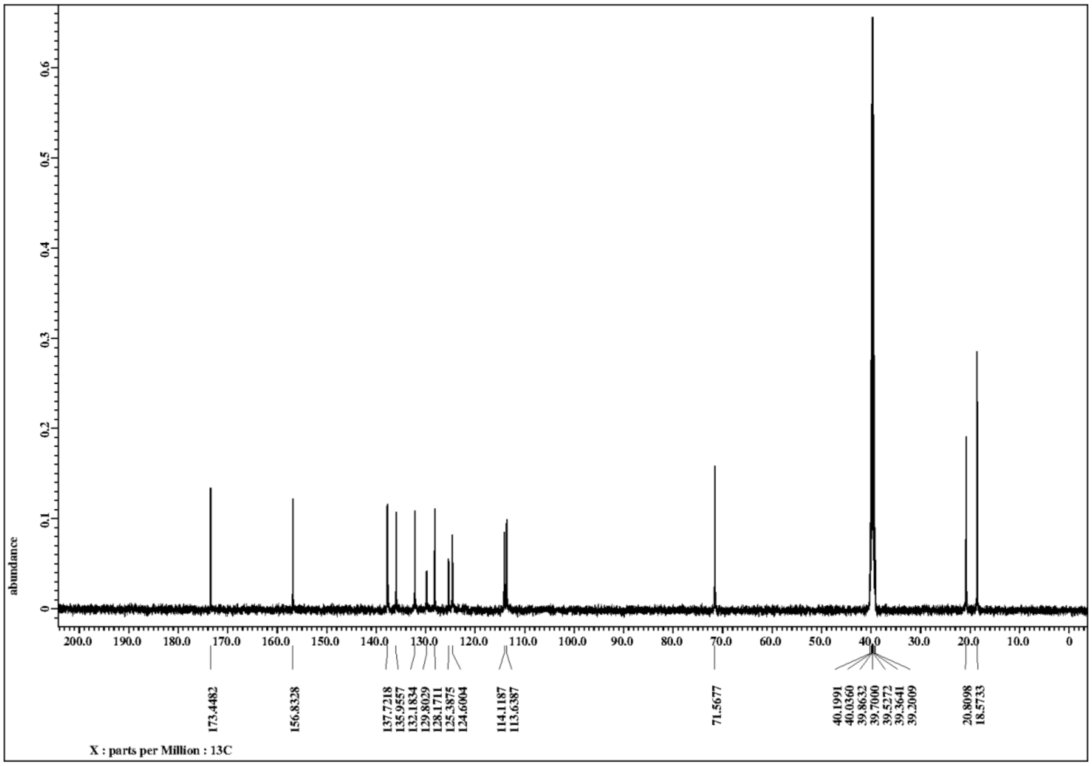

Figure S2. ^{1}H (500 MHz, DMSO-d_6) and ^{13}C NMR (125 MHz, DMSO-d_6) spectra of 1,3,6,8 -tetrakis(4-(α-carboxy–(R)-α-methyl)methoxy-2,6-dimethylphenyl)pyrene, **H_4PLA**.

Solvothermal Synthesis of Zn-PLA

To a solution of **H_4PLA** (100 mg, 0.103 mmol) in 6.5 mL of DMF-H_2O (4:1, *v/v*) mixture was added $Zn(NO_3)_2 \cdot 6H_2O$ (61.2 mg, 0.021 mmol) and 45% aqueous HBF_4 (0.2 mL). The contents were dissolved by sonication, tightly-capped in a glass vial and heated at 90 °C for 2 d. The crystals of **Zn-PLA** were found to develop after cooling down to rt over 2 d, yield 76% (130 mg).

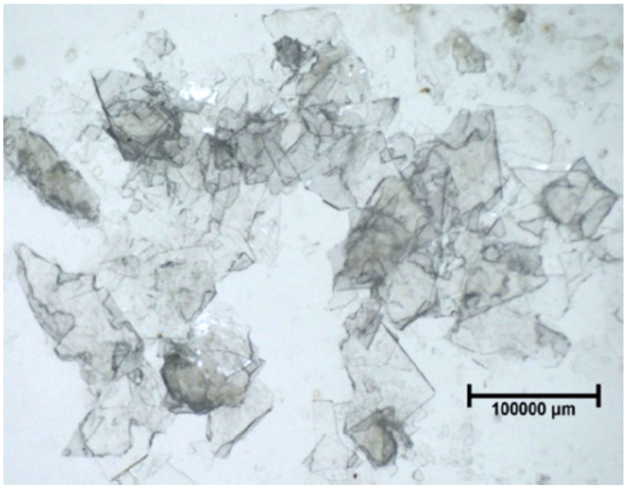

Figure S3. Digital microscopic image of the crystals of pristine **Zn-PLA**.

X-Ray Crystallography

The X-ray diffraction intensity data collection for the crystals of **Zn-PLA** was carried out at 100 K on a Bruker Nonius SMART APEX CCD detector system with Mo-sealed Siemens ceramic diffraction tube (λ = 0.7107 Å) and a highly oriented graphite monochromator operating at 50 kV and 30 mA. The lattice parameters and standard deviations were obtained by a least squares fit using 25 frames with 20 sec/frame exposures with the Bruker APEX2 (Version 2012.10-0) software. The data were collected in a hemisphere mode by phi and omega scans with 2θ = 40º and ~10 sec/frame exposures. Data processing and reduction was done using Bruker

(BrukerAXS Inc, Madison, Wisconsin, USA) SAINT (Version 8.27B) and empirical absorption correction made using Bruker SADABS (Version 2012/1). The structure was solved by Direct Methods using WINGX[1] and SHELXL[2] programs and refined by full matrix least-squares method based on F^2 using ShelxLe 2014 program. Hydrogens were fixed in their ideal geometries, and their contributions included in the refinement. All nonhydrogens were subjected to anisotroic refinement. The topological analysis of the crystal structure of **Zn-PLA** was performed with TOPOS software.[3] The cif for the structure of **Zn-PLA** has been deposited at the Cambridge Crystallographic Data Centre; the deposition number is CCDC 1429649.

References:

1. L. J. Farrugia, *J. Appl. Cryst.*, 2012, **45**, 849–854.

2. G. M. Sheldrick, *Acta Cryst.*, 2008, **A64**, 112–122.

3. V. A. Blatov, A. P. Shevchenko, V. N. Serezhkin, *J. Appl. Cryst.* 2000, **33**, 1193.

Table S1. Crystal data and refinement parameters for **Zn-PLA**.

Parameters	Zn-PLA
Empirical formula	$C_{92}H_{89}NO_{20}Zn_2$
Formula weight	1659.38
Temperature (K)	100(2)
Wavelength (Å)	0.71073
Crystal system	Monoclinic
Space group	*I*2
a (Å)	18.9677(16)
b (Å)	19.9186(17)
c (Å)	31.739(3)
α (°)	90
β (°)	95.287(4)
γ (°)	90
Volume (Å^3)	11940.3(18)
Z	4
Calculated density (mg/m^3)	0.923
Absorption coefficient (mm^{-1})	0.452
F(000)	3472
θ range (°)	2.031 to 28.374
Index ranges	$-25 \leq h \leq 25$<br>$-16 \leq k \leq 26$<br>$-42 \leq l \leq 42$
Reflections collected	51614
Refinement method	Full-matrix least-squares on F^2
Data/restraints/parameters	21469/1/1026
Goodness-of-fit on F^2	0.802
Final R indices [I>2σ(I)]	$R_1 = 0.0871$, $wR_2 = 0.2188$
R indices (all data)	$R_1 = 0.2034$, $wR_2 = 0.2592$
Largest diff. peak and hole e.Å^{-3}	0.425 and -0.411
CCDC deposition number	1429649

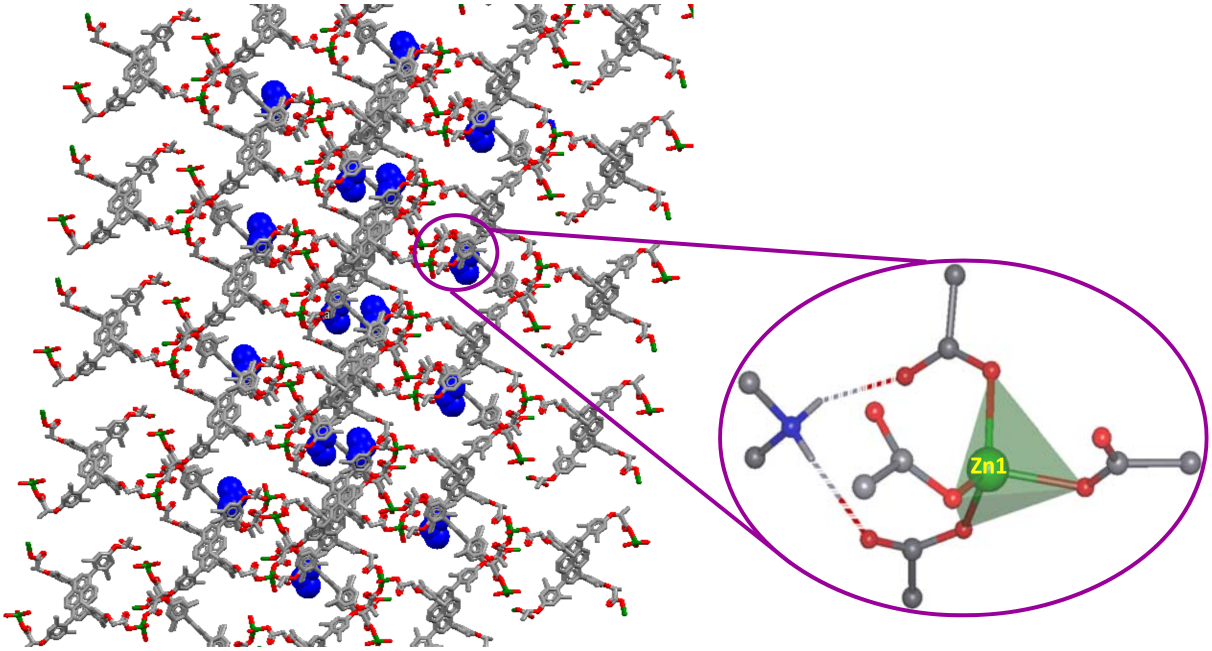

Figure S4. Location of one of the DMA cations (blue) in the voids of **Zn-PLA**. Notice that the ammonium cation is bound by two strong hydrogen bonds with the carboxylate oxygens of **PLA**.

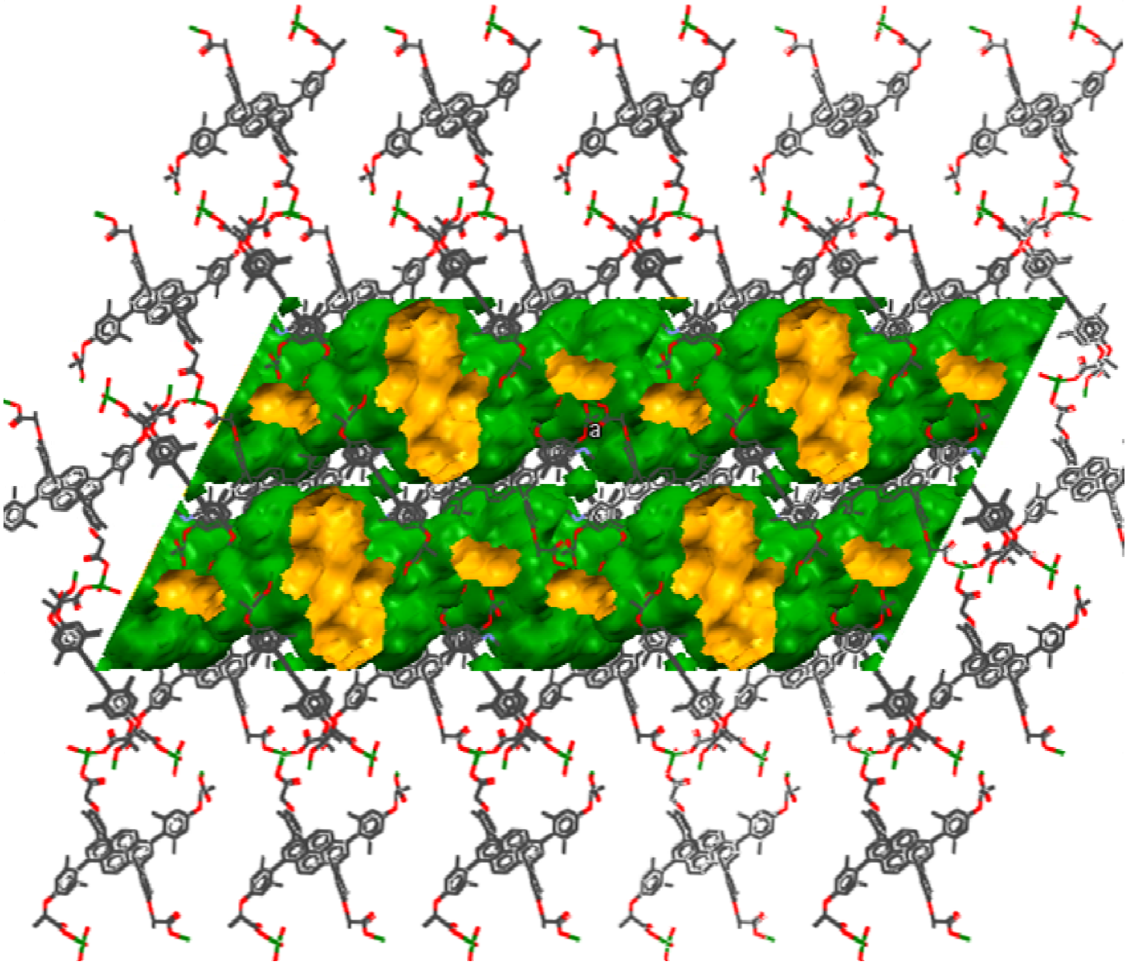

Figure S5. Void volumes in the crystals of **Zn-PLA** as revealed by Mercury with a gridstep of 0.7 Å and probe radius of 1.2 Å. Notice that the voids are largely located near the pyrene fluorophore.

Characterization of Zn-PLA

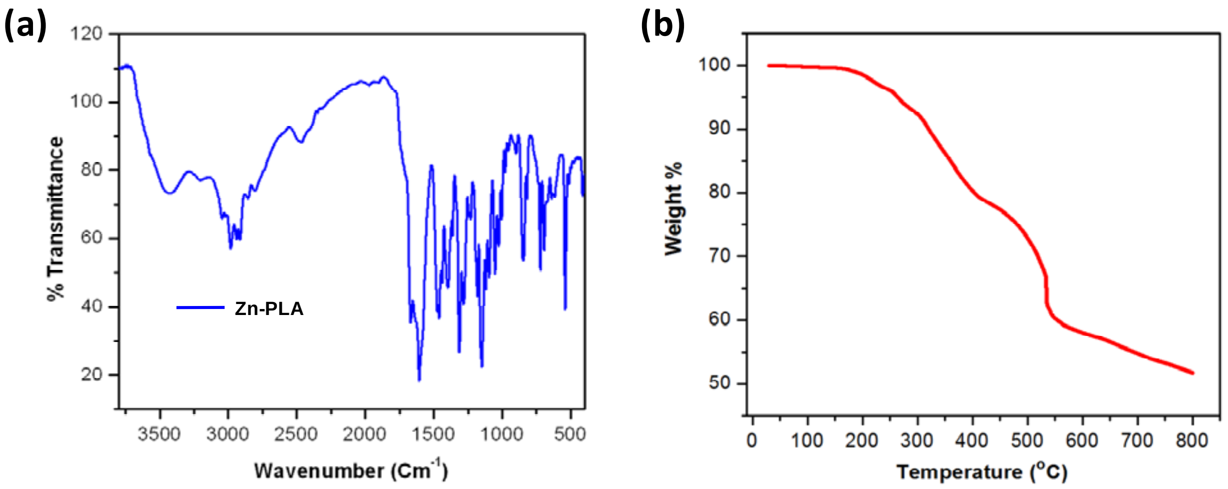

Figure S6. (a) IR spectrum and (b) TGA profile of **Zn-PLA**.

Steady-State Fluorescence Spectroscopy

All the steady-state fluorescence measurements were performed at 298 K on a FluoroMax-4: FM4-3000 spectrofluorometer (Horiba Jobin Yvon Technology), which was standardized with an R928 photomultiplier tube, a DM302 photon-counting acquisition module biased at 950 V, a 1200 lines/mm grating blazed at 330 nm in the excitation monochromator and a 1200 lines/mm grating blazed at 500 nm in the emission monochromator. The slit widths for both excitation and emission were fixed at 1.0 nm bandpass and the accuracy in measuring the wavelength was ±2.0 nm. The solid-state fluorescence quantum yield determinations were performed by using a 'Quanta-φ: F-3029' Horiba Scientific integrating sphere (sphere inner diameter: 150 mm) connected to the spectrofluorimeter.

Fluorescence Quenching Titration Experiments with Zn-PLA

The fluorescence quenching studies were performed by using an aqueous suspension of **Zn-PLA** at 298 K. Typically, the suspension was prepared by adding the crystals of **Zn-PLA** (1.5 mg) into 3 mL of water (HPLC grade) followed by sonication for 10 min. The resultant suspension of MOF was subsequently placed in a quartz cell of 1 cm width. The fluorescence titrations were carried out for excitation at 350 nm by gradually adding the aqueous solutions of various analytes to the suspension of **Zn-PLA**. The suspension was stirred magnetically within the spectrometer during the acquisition of fluorescence. Each titration was repeated at least three times to obtain concordant values.

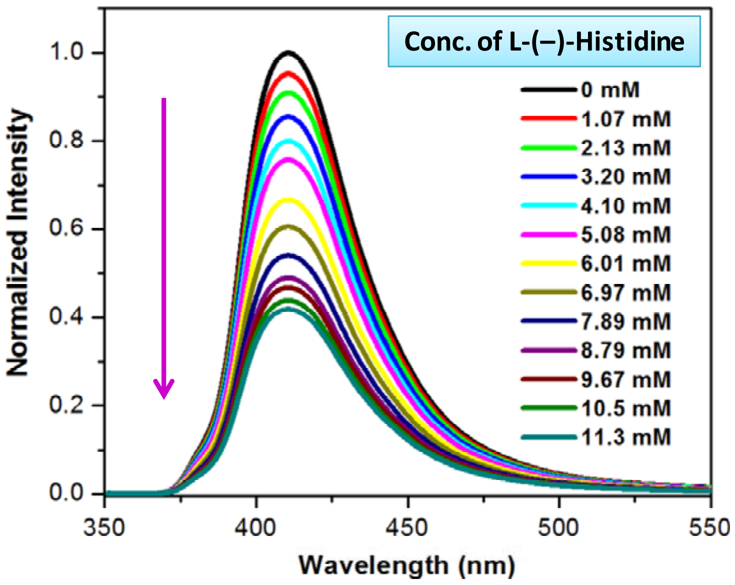

Figure S7. Quenching of fluorescence intensity of **Zn-PLA** with increasing concentration of L-(–)-histidine in water (λ_{ex} = 350 nm).

Fluorescence Quenching Experiments with PTE

Due to the solubility issues with **PTE** in DMSO, a stock solution (5×10^{-4} M) was prepared in 1,4-dioxane. Using this solution, PTE solution of 1 μM concentration was prepared by diluting 20 μL of the stock solution with a 10 mL of DMSO. The fluorescence lifetime decay of **PTE** was recorded using time-correlated single photon counting instrument (Fluorolog, Horiba Jobin Yvon) using a picosecond pulsed diode laser (λ_{ex} = 375 nm) for excitation. The instrument response function was measured by using a scatterer and the decay was fitted by using a reconvolution software supplied with the instrument. The fluorescence decay of **PTE** (λ_{em} = 410 nm) was best fitted to a biexponential function with the components: τ_1 = 1.18 ns (α_1 = 0.026) and τ_2 = 18.73 ns (α_2 = 0.974). Thus, the average fluorescence lifetime of **PTE** turns out to be 18.3 ns using the formula: $\tau = \frac{(\alpha_1\tau_1 + \alpha_2\tau_2)}{(\alpha_1 + \alpha_2)}$. The fluorescence quenching studies with **PTE** (1 μM in DMSO) were performed in the presence of increasing concentrations of aqueous solutions of

various amino acids. A representative plot for the quenching of fluorescence of **PTE** by tyrosine is shown in Figure S9. The bimolecular quenching rate constants (k_q) were calculated from the corresponding Stern-Volmer constants (K_{SV}) using the formula: $K_{SV} = k_q\tau$.

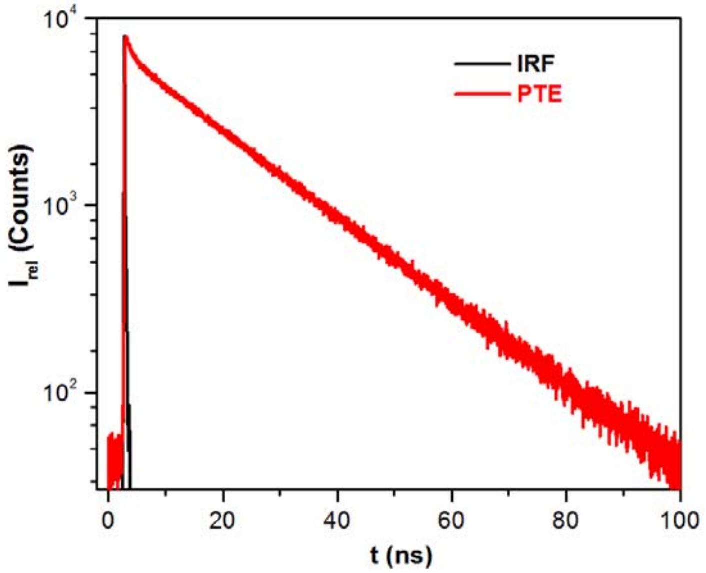

Figure S8. Fluorescence lifetime decay trace (λ_{ex} = 375 nm; λ_{em} = 410 nm) of **PTE** in DMSO (1 μM).

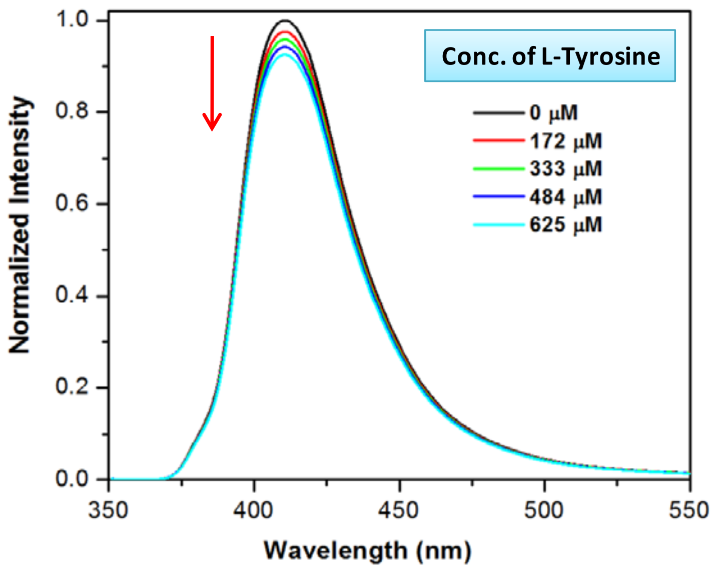

Figure S9. Quenching of fluorescence intensity of **PTE** (1 μM) in DMSO (λ_{ex} = 350 nm; λ_{em} = 410 nm) with increasing concentrations of aqueous L-tyrosine.

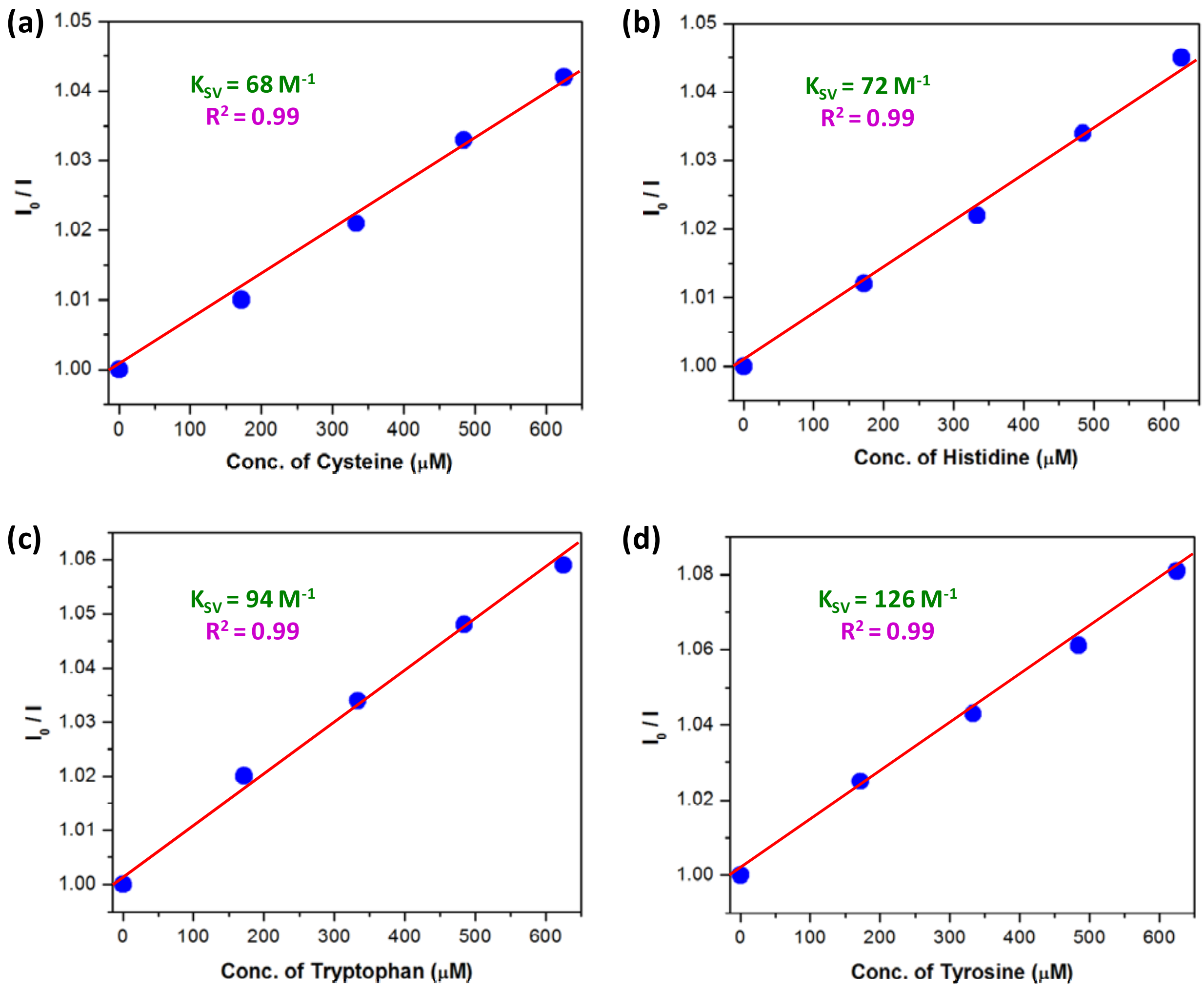

Figure S10. Determination of Stern-Volmer constants of various L-amino acids for the quenching of fluorescence of **PTE** (1 μM) in DMSO (λ_{ex} = 350 nm; λ_{em} = 410 nm).

Table S2. Quenching rate data of **PTE** in DMSO obtained from Stern-Volmer plots for various amino acid quenchers.

**L-Amino acid**	K_{SV} **(M^{-1})**	k_q **(M^{-1} s^{-1})**
Cysteine	68	3.7×10^9
Histidine	72	3.9×10^9
Tryptophan	94	5.1×10^9
Tyrosine	126	6.9×10^9

Postsynthetic Dye Exchange Studies

To substantiate the exchange of cationic dimethylammonium species inside the voids of **Zn-PLA** MOF, dye-exchange studies were performed. For this purpose, the colorless crystals of the **Zn-PLA** MOF were dispersed in a ca. 10^{-5} M solution of methylene blue (a cationic dye), bromophenol blue (an anionic dye) and nile red (a neutral dye) in DMF. Gradual adsorption of the cationic dye by **Zn-PLA** with time was monitored by UV-vis absorption spectra for changes in the absorption of the supernatant solution, cf. Figure S11. The dye exchange process could be gradually made out from progressive decrease in the absorption of the dye solution in conjunction with blue coloration of the initially colorless crystals of **Zn-PLA**. Interestingly, the dye-exchange was not observed for the anionic bromophenol blue or neutral nile red dye, attesting to the fact that the cationic dimethylammonium species undergo exchange with only cationic dye.

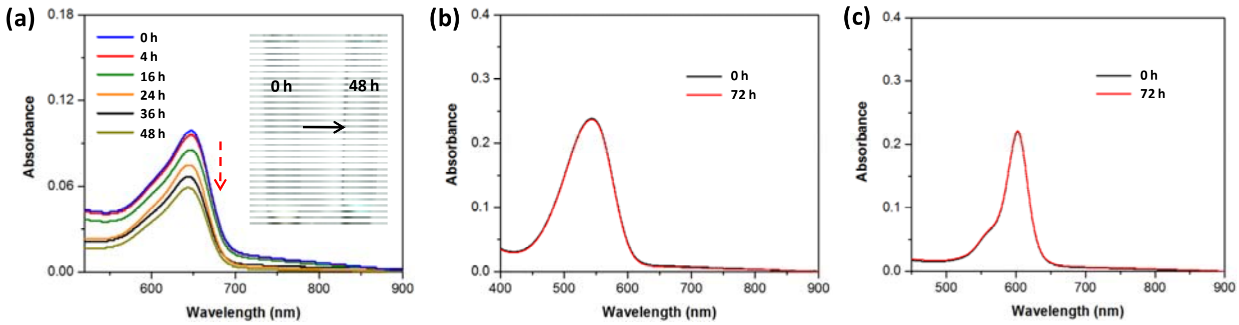

Figure S11. Changes in the UV-vis adsorption spectra of DMF solution of methylene blue (a), bromophenol blue (b) and nile red (c) in the presence of added crystals of **Zn-PLA**. Notice the diminution in absorption of methylene blue (cationic dye) solution with time, as the dye is gradually adsorbed by **Zn-PLA**. The inset shows coloration of the MOF crystals with time.

Table S3. Atomic coordinates (x 10^4) and equivalent isotropic displacement parameters ($Å^2$x 10^3) for **Zn-PLA**. U(eq) is defined as one third of the trace of the orthogonalized U^{ij} tensor.

	x	y	z	U(eq)
Zn(1)	3582(1)	-1410(1)	4085(1)	84(1)
Zn(2)	11439(3)	889(3)	1748(1)	313(3)
O(1)	4258(4)	-1776(6)	3323(3)	152(5)
O(2)	4625(4)	-1587(4)	3957(3)	124(3)
O(3)	5607(3)	-1852(4)	3137(3)	96(2)
O(4)	2383(4)	2713(5)	372(2)	112(3)
O(5)	1672(4)	2600(5)	896(2)	114(3)
O(6)	2922(3)	1491(4)	508(2)	89(2)
O(7)	8828(5)	3992(5)	-351(3)	116(3)
O(8)	7780(6)	4452(4)	-315(3)	111(3)
O(9)	8192(5)	4784(5)	544(2)	139(4)
O(10)	11484(5)	944(7)	2354(4)	170(5)
O(11)	11130(5)	1996(8)	2426(3)	167(5)
O(12)	10760(4)	1830(5)	3242(2)	121(3)
O(13)	3098(4)	-812(5)	3692(2)	114(3)
O(14)	2087(5)	-1368(6)	3788(3)	148(3)
O(15)	1252(4)	-422(4)	3330(2)	100(2)
O(16)	2280(5)	4739(7)	3138(3)	158(2)
O(17)	2845(6)	5540(6)	3461(3)	158(2)
O(18)	952(6)	5254(6)	3186(3)	158(2)
O(19)	11419(5)	1824(4)	1629(3)	135(3)
O(20)	10719(10)	798(17)	1632(10)	417(19)
C(1)	4696(8)	-1737(8)	3602(4)	121(5)
C(2)	5441(5)	-2013(6)	3554(4)	98(4)
C(3)	5484(7)	-2794(7)	3584(4)	138(5)
C(4)	5904(4)	-1260(6)	3067(4)	86(4)
C(5)	5823(5)	-679(5)	3310(3)	65(2)
C(6)	6122(5)	-95(5)	3195(3)	68(2)
C(7)	6485(4)	-70(5)	2830(3)	58(2)
C(8)	6551(5)	-637(5)	2573(3)	66(2)

C(9)	6231(5)	-1196(6)	2685(3)	85(3)
C(10)	6116(7)	537(7)	3467(3)	114(4)
C(11)	6935(6)	-637(5)	2182(2)	85(3)
C(12)	11310(9)	1493(11)	2599(6)	136(6)
C(13)	11278(7)	1404(10)	3053(5)	158(7)
C(14)	12001(7)	1610(9)	3256(5)	160(6)
C(15)	10064(5)	1626(8)	3109(3)	100(5)
C(16)	9901(7)	1103(8)	2848(4)	104(5)
C(17)	9195(5)	926(5)	2718(3)	74(3)
C(18)	8678(4)	1376(6)	2874(3)	75(3)
C(19)	8844(5)	1921(5)	3135(2)	71(3)
C(20)	9547(4)	2025(6)	3248(2)	80(3)
C(21)	9013(7)	356(7)	2401(3)	111(4)
C(22)	8280(4)	2335(5)	3273(3)	71(3)
C(23)	8378(9)	4334(11)	-173(4)	134(7)
C(24)	8710(7)	4653(9)	242(4)	148(7)
C(25)	7835(6)	4240(7)	731(3)	88(4)
C(26)	7923(4)	3630(7)	592(3)	85(3)
C(27)	7524(4)	3088(5)	780(3)	66(3)
C(28)	7090(4)	3250(5)	1099(2)	48(2)
C(29)	7039(4)	3909(6)	1219(3)	71(3)
C(30)	7431(5)	4361(6)	1033(3)	78(3)
C(31)	7589(6)	2339(7)	638(4)	118(4)
C(32)	6621(6)	4105(5)	1590(3)	84(3)
C(33)	2096(6)	2395(6)	633(3)	76(3)
C(34)	2299(4)	1677(7)	690(3)	89(4)
C(35)	1750(6)	1217(10)	454(5)	168(7)
C(36)	3583(5)	1643(5)	714(3)	64(3)
C(37)	4135(5)	1383(5)	571(3)	77(3)
C(38)	4797(4)	1497(5)	768(3)	61(2)
C(39)	4863(4)	1946(4)	1108(2)	52(2)
C(40)	4267(5)	2219(5)	1259(2)	63(2)
C(41)	3608(5)	2056(6)	1063(3)	84(3)
C(42)	5439(5)	1175(6)	592(3)	110(4)
C(43)	4343(5)	2714(5)	1659(3)	81(3)
C(44)	6862(5)	596(4)	2693(2)	59(2)

C(45)	7536(4)	731(4)	2855(2)	52(2)
C(46)	7905(4)	1239(4)	2688(2)	54(2)
C(47)	7627(4)	1651(5)	2354(2)	54(2)
C(48)	8009(4)	2134(5)	2152(2)	58(2)
C(49)	7727(4)	2495(4)	1830(2)	58(2)
C(50)	7001(3)	2374(4)	1653(2)	42(2)
C(51)	6693(4)	2737(4)	1287(2)	50(2)
C(52)	6007(4)	2587(5)	1145(2)	56(2)
C(53)	5589(4)	2073(4)	1320(2)	52(2)
C(54)	5880(4)	1737(4)	1666(2)	53(2)
C(55)	5518(4)	1222(4)	1856(2)	61(2)
C(56)	5809(4)	856(4)	2184(3)	58(2)
C(57)	6536(4)	979(4)	2355(2)	52(2)
C(58)	6911(4)	1467(4)	2196(2)	50(2)
C(59)	6596(4)	1888(4)	1836(2)	51(2)
C(60)	2436(10)	-931(9)	3601(5)	136(6)
C(61)	2043(8)	-399(7)	3295(3)	124(5)
C(62)	2148(9)	-650(12)	2839(4)	226(11)
C(63)	1075(6)	-57(5)	3676(3)	75(3)
C(64)	1551(6)	176(5)	3993(3)	73(3)
C(65)	1275(6)	549(5)	4328(3)	78(3)
C(66)	553(4)	673(4)	4319(2)	48(2)
C(67)	82(5)	409(4)	3981(3)	63(2)
C(68)	346(6)	32(5)	3684(3)	73(3)
C(69)	0	721(6)	5000	49(3)
C(70)	261(4)	1068(5)	4667(3)	61(2)
C(71)	-693(5)	561(5)	3973(2)	72(3)
C(72)	1823(6)	846(6)	4682(3)	98(4)
C(73)	270(4)	1800(4)	4663(2)	44(2)
C(74)	0	2116(6)	5000	55(3)
C(75)	0	2835(6)	5000	44(3)
C(76)	240(4)	3214(4)	4650(2)	49(2)
C(77)	479(4)	2800(5)	4318(3)	58(2)
C(78)	494(4)	2147(5)	4312(3)	59(2)
C(79)	0	4207(8)	5000	73(4)
C(80)	229(4)	3914(5)	4657(3)	57(2)

C(81)	432(5)	4262(5)	4282(3)	64(2)
C(82)	-31(5)	4349(5)	3923(3)	69(3)
C(83)	149(6)	4637(5)	3546(3)	88(3)
C(84)	757(7)	4957(5)	3552(3)	83(3)
C(85)	1241(7)	4940(6)	3914(4)	101(4)
C(86)	1093(5)	4626(5)	4278(3)	69(2)
C(87)	1614(6)	4565(7)	4696(4)	114(4)
C(88)	-759(6)	4022(7)	3933(4)	128(5)
C(94)	8977(9)	5389(10)	158(4)	192(9)
C(95)	1508(8)	6052(9)	2774(4)	158(2)
C(96)	2170(11)	5400(12)	3215(5)	158(2)
C(97)	1517(9)	5693(10)	3186(4)	158(2)
N(1)	1557(5)	8670(8)	4630(3)	137(4)
C(92)	1346(7)	8989(11)	5013(4)	182(9)
C(93)	860(8)	8646(12)	4348(4)	185(7)

Table S4. Bond lengths [Å] and angles [°] for **Zn-PLA**.

Zn(1)-O(13)	1.900(8)
Zn(1)-O(7)#1	1.976(9)
Zn(1)-O(5)#2	2.031(10)
Zn(1)-O(2)	2.086(8)
Zn(2)-O(20)	1.39(2)
Zn(2)-O(17)#3	1.714(9)
Zn(2)-O(19)	1.899(9)
Zn(2)-O(10)	1.921(13)
O(1)-C(1)	1.159(12)
O(2)-C(1)	1.186(13)
O(3)-C(4)	1.333(12)
O(3)-C(2)	1.426(12)
O(4)-C(33)	1.212(12)
O(5)-C(33)	1.279(12)
O(5)-Zn(1)#4	2.031(10)
O(6)-C(36)	1.392(10)
O(6)-C(34)	1.413(10)
O(7)-C(23)	1.266(18)
O(7)-Zn(1)#5	1.976(9)
O(8)-C(23)	1.203(17)
O(9)-C(25)	1.435(13)
O(9)-C(24)	1.457(14)
O(10)-C(12)	1.398(19)
O(11)-C(12)	1.178(17)
O(12)-C(15)	1.408(11)
O(12)-C(13)	1.469(17)
O(13)-C(60)	1.284(17)
O(14)-C(60)	1.274(16)
O(15)-C(63)	1.383(11)
O(15)-C(61)	1.517(14)
O(16)-C(96)	1.36(2)
O(17)-C(96)	1.46(2)
O(17)-Zn(2)#6	1.714(9)
O(18)-C(97)	1.383(18)

O(18)-C(84)	1.386(12)
C(1)-C(2)	1.536(17)
C(2)-C(3)	1.561(17)
C(2)-H(2)	0.9800
C(3)-H(3A)	0.9600
C(3)-H(3B)	0.9600
C(3)-H(3C)	0.9600
C(4)-C(5)	1.407(15)
C(4)-C(9)	1.417(13)
C(5)-C(6)	1.358(13)
C(5)-H(5)	0.9300
C(6)-C(7)	1.401(11)
C(6)-C(10)	1.528(15)
C(7)-C(8)	1.405(12)
C(7)-C(44)	1.586(12)
C(8)-C(9)	1.333(12)
C(8)-C(11)	1.496(12)
C(9)-H(9)	0.9300
C(10)-H(10A)	0.9600
C(10)-H(10B)	0.9600
C(10)-H(10C)	0.9600
C(11)-H(11A)	0.9600
C(11)-H(11B)	0.9600
C(11)-H(11C)	0.9600
C(12)-C(13)	1.46(2)
C(13)-C(14)	1.517(19)
C(13)-H(13)	0.9800
C(14)-H(14A)	0.9600
C(14)-H(14B)	0.9600
C(14)-H(14C)	0.9600
C(15)-C(16)	1.349(17)
C(15)-C(20)	1.366(17)
C(16)-C(17)	1.409(16)
C(16)-H(16)	0.9300
C(17)-C(18)	1.449(13)
C(17)-C(21)	1.535(15)

C(18)-C(19)	1.385(14)
C(18)-C(46)	1.554(12)
C(19)-C(20)	1.366(11)
C(19)-C(22)	1.449(12)
C(20)-H(20)	0.9300
C(21)-H(21A)	0.9600
C(21)-H(21B)	0.9600
C(21)-H(21C)	0.9600
C(22)-H(22A)	0.9600
C(22)-H(22B)	0.9600
C(22)-H(22C)	0.9600
C(23)-C(24)	1.544(16)
C(24)-C(94)	1.58(2)
C(24)-H(24)	0.9800
C(25)-C(30)	1.304(14)
C(25)-C(26)	1.308(16)
C(26)-C(27)	1.476(13)
C(26)-H(26)	0.9300
C(27)-C(28)	1.400(11)
C(27)-C(31)	1.567(15)
C(28)-C(29)	1.371(12)
C(28)-C(51)	1.433(11)
C(29)-C(30)	1.337(13)
C(29)-C(32)	1.531(12)
C(30)-H(30)	0.9300
C(31)-H(31A)	0.9600
C(31)-H(31B)	0.9600
C(31)-H(31C)	0.9600
C(32)-H(32A)	0.9600
C(32)-H(32B)	0.9600
C(32)-H(32C)	0.9600
C(33)-C(34)	1.488(15)
C(34)-C(35)	1.530(16)
C(34)-H(34)	0.9800
C(35)-H(35A)	0.9600
C(35)-H(35B)	0.9600

C(35)-H(35C)	0.9600
C(36)-C(37)	1.289(12)
C(36)-C(41)	1.377(13)
C(37)-C(38)	1.369(12)
C(37)-H(37)	0.9300
C(38)-C(39)	1.398(11)
C(38)-C(42)	1.528(12)
C(39)-C(40)	1.379(11)
C(39)-C(53)	1.497(10)
C(40)-C(41)	1.383(12)
C(40)-C(43)	1.603(12)
C(41)-H(41)	0.9300
C(42)-H(42A)	0.9600
C(42)-H(42B)	0.9600
C(42)-H(42C)	0.9600
C(43)-H(43A)	0.9600
C(43)-H(43B)	0.9600
C(43)-H(43C)	0.9600
C(44)-C(45)	1.359(10)
C(44)-C(57)	1.412(10)
C(45)-C(46)	1.365(11)
C(45)-H(45)	0.9300
C(46)-C(47)	1.404(10)
C(47)-C(48)	1.395(12)
C(47)-C(58)	1.450(10)
C(48)-C(49)	1.321(10)
C(48)-H(48)	0.9300
C(49)-C(50)	1.460(10)
C(49)-H(49)	0.9300
C(50)-C(59)	1.395(10)
C(50)-C(51)	1.443(9)
C(51)-C(52)	1.370(10)
C(52)-C(53)	1.437(11)
C(52)-H(52)	0.9300
C(53)-C(54)	1.359(10)
C(54)-C(55)	1.401(11)

C(54)-C(59)	1.447(10)
C(55)-C(56)	1.347(10)
C(55)-H(55)	0.9300
C(56)-C(57)	1.456(11)
C(56)-H(56)	0.9300
C(57)-C(58)	1.331(11)
C(58)-C(59)	1.498(10)
C(60)-C(61)	1.58(2)
C(61)-C(62)	1.559(16)
C(61)-H(61)	0.9800
C(62)-H(62A)	0.9600
C(62)-H(62B)	0.9600
C(62)-H(62C)	0.9600
C(63)-C(64)	1.368(13)
C(63)-C(68)	1.398(13)
C(64)-C(65)	1.434(13)
C(64)-H(64)	0.9300
C(65)-C(66)	1.390(12)
C(65)-C(72)	1.575(13)
C(66)-C(67)	1.431(11)
C(66)-C(70)	1.505(11)
C(67)-C(68)	1.335(11)
C(67)-C(71)	1.500(12)
C(68)-H(68)	0.9300
C(69)-C(70)#7	1.390(10)
C(69)-C(70)	1.390(10)
C(69)-H(69)	0.9300
C(70)-C(73)	1.458(11)
C(71)-H(71A)	0.9600
C(71)-H(71B)	0.9600
C(71)-H(71C)	0.9600
C(72)-H(72A)	0.9600
C(72)-H(72B)	0.9600
C(72)-H(72C)	0.9600
C(73)-C(74)	1.380(9)
C(73)-C(78)	1.410(11)

C(74)-C(73)#7	1.380(9)
C(74)-C(75)	1.431(15)
C(75)-C(76)	1.451(10)
C(75)-C(76)#7	1.451(10)
C(76)-C(80)	1.395(10)
C(76)-C(77)	1.442(11)
C(77)-C(78)	1.301(11)
C(77)-H(77)	0.9300
C(78)-H(78)	0.9300
C(79)-C(80)	1.344(10)
C(79)-C(80)#7	1.344(10)
C(79)-H(79)	0.9300
C(80)-C(81)	1.458(12)
C(81)-C(82)	1.383(12)
C(81)-C(86)	1.451(12)
C(82)-C(83)	1.397(12)
C(82)-C(88)	1.529(14)
C(83)-C(84)	1.316(14)
C(83)-H(83)	0.9300
C(84)-C(85)	1.403(15)
C(85)-C(86)	1.365(13)
C(85)-H(85)	0.9300
C(86)-C(87)	1.585(15)
C(87)-H(87A)	0.9600
C(87)-H(87B)	0.9600
C(87)-H(87C)	0.9600
C(88)-H(88A)	0.9600
C(88)-H(88B)	0.9600
C(88)-H(88C)	0.9600
C(94)-H(94A)	0.9600
C(94)-H(94B)	0.9600
C(94)-H(94C)	0.9600
C(95)-C(97)	1.487(18)
C(95)-H(95A)	0.9600
C(95)-H(95B)	0.9600
C(95)-H(95C)	0.9600

C(96)-C(97)	1.37(2)
C(97)-H(97)	0.9800
N(1)-C(92)	1.459(15)
N(1)-C(93)	1.529(16)
N(1)-H(1A)	0.8900
N(1)-H(1B)	0.8899
C(92)-H(92A)	0.9600
C(92)-H(92B)	0.9600
C(92)-H(92C)	0.9600
C(93)-H(93A)	0.9600
C(93)-H(93B)	0.9600
C(93)-H(93C)	0.9600

O(13)-Zn(1)-O(7)#1	113.5(4)
O(13)-Zn(1)-O(5)#2	121.8(4)
O(7)#1-Zn(1)-O(5)#2	113.8(3)
O(13)-Zn(1)-O(2)	112.8(4)
O(7)#1-Zn(1)-O(2)	95.4(4)
O(5)#2-Zn(1)-O(2)	94.2(3)
O(20)-Zn(2)-O(17)#3	129.6(13)
O(20)-Zn(2)-O(19)	94.3(15)
O(17)#3-Zn(2)-O(19)	108.9(5)
O(20)-Zn(2)-O(10)	103.0(12)
O(17)#3-Zn(2)-O(10)	116.6(5)
O(19)-Zn(2)-O(10)	98.1(6)
C(1)-O(2)-Zn(1)	114.8(10)
C(4)-O(3)-C(2)	119.1(10)
C(33)-O(5)-Zn(1)#4	119.6(7)
C(36)-O(6)-C(34)	120.2(7)
C(23)-O(7)-Zn(1)#5	120.7(9)
C(25)-O(9)-C(24)	120.6(11)
C(12)-O(10)-Zn(2)	127.7(11)
C(15)-O(12)-C(13)	110.7(12)
C(60)-O(13)-Zn(1)	115.8(11)
C(63)-O(15)-C(61)	110.9(9)
C(96)-O(17)-Zn(2)#6	123.8(9)

C(97)-O(18)-C(84)	122.5(11)
O(1)-C(1)-O(2)	127.4(16)
O(1)-C(1)-C(2)	120.0(13)
O(2)-C(1)-C(2)	111.7(12)
O(3)-C(2)-C(1)	107.2(9)
O(3)-C(2)-C(3)	105.5(9)
C(1)-C(2)-C(3)	113.3(10)
O(3)-C(2)-H(2)	110.2
C(1)-C(2)-H(2)	110.2
C(3)-C(2)-H(2)	110.2
C(2)-C(3)-H(3A)	109.5
C(2)-C(3)-H(3B)	109.5
H(3A)-C(3)-H(3B)	109.5
C(2)-C(3)-H(3C)	109.5
H(3A)-C(3)-H(3C)	109.5
H(3B)-C(3)-H(3C)	109.5
O(3)-C(4)-C(5)	124.6(9)
O(3)-C(4)-C(9)	116.5(12)
C(5)-C(4)-C(9)	118.1(10)
C(6)-C(5)-C(4)	119.2(8)
C(6)-C(5)-H(5)	120.4
C(4)-C(5)-H(5)	120.4
C(5)-C(6)-C(7)	120.2(9)
C(5)-C(6)-C(10)	121.9(9)
C(7)-C(6)-C(10)	117.9(9)
C(6)-C(7)-C(8)	121.8(8)
C(6)-C(7)-C(44)	121.6(9)
C(8)-C(7)-C(44)	116.5(7)
C(9)-C(8)-C(7)	116.7(8)
C(9)-C(8)-C(11)	119.4(10)
C(7)-C(8)-C(11)	123.9(8)
C(8)-C(9)-C(4)	123.4(11)
C(8)-C(9)-H(9)	118.3
C(4)-C(9)-H(9)	118.3
C(6)-C(10)-H(10A)	109.5
C(6)-C(10)-H(10B)	109.5

H(10A)-C(10)-H(10B)	109.5
C(6)-C(10)-H(10C)	109.5
H(10A)-C(10)-H(10C)	109.5
H(10B)-C(10)-H(10C)	109.5
C(8)-C(11)-H(11A)	109.5
C(8)-C(11)-H(11B)	109.5
H(11A)-C(11)-H(11B)	109.5
C(8)-C(11)-H(11C)	109.5
H(11A)-C(11)-H(11C)	109.5
H(11B)-C(11)-H(11C)	109.5
O(11)-C(12)-O(10)	118.6(17)
O(11)-C(12)-C(13)	121.7(18)
O(10)-C(12)-C(13)	119.2(18)
C(12)-C(13)-O(12)	115.2(13)
C(12)-C(13)-C(14)	105.6(12)
O(12)-C(13)-C(14)	106.4(15)
C(12)-C(13)-H(13)	109.8
O(12)-C(13)-H(13)	109.8
C(14)-C(13)-H(13)	109.8
C(13)-C(14)-H(14A)	109.5
C(13)-C(14)-H(14B)	109.5
H(14A)-C(14)-H(14B)	109.5
C(13)-C(14)-H(14C)	109.5
H(14A)-C(14)-H(14C)	109.5
H(14B)-C(14)-H(14C)	109.5
C(16)-C(15)-C(20)	121.2(9)
C(16)-C(15)-O(12)	124.3(14)
C(20)-C(15)-O(12)	114.5(13)
C(15)-C(16)-C(17)	122.1(11)
C(15)-C(16)-H(16)	119.0
C(17)-C(16)-H(16)	119.0
C(16)-C(17)-C(18)	113.6(11)
C(16)-C(17)-C(21)	121.9(11)
C(18)-C(17)-C(21)	124.2(9)
C(19)-C(18)-C(17)	124.5(9)
C(19)-C(18)-C(46)	121.3(8)

C(17)-C(18)-C(46)	114.0(10)
C(20)-C(19)-C(18)	116.0(10)
C(20)-C(19)-C(22)	124.4(9)
C(18)-C(19)-C(22)	119.6(7)
C(19)-C(20)-C(15)	122.6(10)
C(19)-C(20)-H(20)	118.7
C(15)-C(20)-H(20)	118.7
C(17)-C(21)-H(21A)	109.5
C(17)-C(21)-H(21B)	109.5
H(21A)-C(21)-H(21B)	109.5
C(17)-C(21)-H(21C)	109.5
H(21A)-C(21)-H(21C)	109.5
H(21B)-C(21)-H(21C)	109.5
C(19)-C(22)-H(22A)	109.5
C(19)-C(22)-H(22B)	109.5
H(22A)-C(22)-H(22B)	109.5
C(19)-C(22)-H(22C)	109.5
H(22A)-C(22)-H(22C)	109.5
H(22B)-C(22)-H(22C)	109.5
O(8)-C(23)-O(7)	126.0(12)
O(8)-C(23)-C(24)	122.4(17)
O(7)-C(23)-C(24)	111.3(15)
O(9)-C(24)-C(23)	112.8(10)
O(9)-C(24)-C(94)	101.0(13)
C(23)-C(24)-C(94)	110.4(12)
O(9)-C(24)-H(24)	110.8
C(23)-C(24)-H(24)	110.8
C(94)-C(24)-H(24)	110.8
C(30)-C(25)-C(26)	121.4(11)
C(30)-C(25)-O(9)	119.8(13)
C(26)-C(25)-O(9)	118.8(10)
C(25)-C(26)-C(27)	117.2(10)
C(25)-C(26)-H(26)	121.4
C(27)-C(26)-H(26)	121.4
C(28)-C(27)-C(26)	118.8(10)
C(28)-C(27)-C(31)	119.7(9)

C(26)-C(27)-C(31)	121.5(10)
C(29)-C(28)-C(27)	118.8(8)
C(29)-C(28)-C(51)	121.0(8)
C(27)-C(28)-C(51)	120.2(8)
C(30)-C(29)-C(28)	117.8(9)
C(30)-C(29)-C(32)	121.0(11)
C(28)-C(29)-C(32)	120.8(9)
C(25)-C(30)-C(29)	125.9(11)
C(25)-C(30)-H(30)	117.0
C(29)-C(30)-H(30)	117.0
C(27)-C(31)-H(31A)	109.5
C(27)-C(31)-H(31B)	109.5
H(31A)-C(31)-H(31B)	109.5
C(27)-C(31)-H(31C)	109.5
H(31A)-C(31)-H(31C)	109.5
H(31B)-C(31)-H(31C)	109.5
C(29)-C(32)-H(32A)	109.5
C(29)-C(32)-H(32B)	109.5
H(32A)-C(32)-H(32B)	109.5
C(29)-C(32)-H(32C)	109.5
H(32A)-C(32)-H(32C)	109.5
H(32B)-C(32)-H(32C)	109.5
O(4)-C(33)-O(5)	129.1(12)
O(4)-C(33)-C(34)	117.2(11)
O(5)-C(33)-C(34)	113.5(10)
O(6)-C(34)-C(33)	114.8(10)
O(6)-C(34)-C(35)	101.5(9)
C(33)-C(34)-C(35)	111.2(10)
O(6)-C(34)-H(34)	109.7
C(33)-C(34)-H(34)	109.7
C(35)-C(34)-H(34)	109.7
C(34)-C(35)-H(35A)	109.5
C(34)-C(35)-H(35B)	109.5
H(35A)-C(35)-H(35B)	109.5
C(34)-C(35)-H(35C)	109.5
H(35A)-C(35)-H(35C)	109.5

H(35B)-C(35)-H(35C)	109.5
C(37)-C(36)-C(41)	123.7(8)
C(37)-C(36)-O(6)	118.2(9)
C(41)-C(36)-O(6)	118.1(9)
C(36)-C(37)-C(38)	120.8(9)
C(36)-C(37)-H(37)	119.6
C(38)-C(37)-H(37)	119.6
C(37)-C(38)-C(39)	118.2(8)
C(37)-C(38)-C(42)	119.4(9)
C(39)-C(38)-C(42)	122.2(8)
C(40)-C(39)-C(38)	120.1(7)
C(40)-C(39)-C(53)	121.8(8)
C(38)-C(39)-C(53)	117.8(8)
C(39)-C(40)-C(41)	119.1(8)
C(39)-C(40)-C(43)	120.0(7)
C(41)-C(40)-C(43)	120.9(8)
C(36)-C(41)-C(40)	117.8(8)
C(36)-C(41)-H(41)	121.1
C(40)-C(41)-H(41)	121.1
C(38)-C(42)-H(42A)	109.5
C(38)-C(42)-H(42B)	109.5
H(42A)-C(42)-H(42B)	109.5
C(38)-C(42)-H(42C)	109.5
H(42A)-C(42)-H(42C)	109.5
H(42B)-C(42)-H(42C)	109.5
C(40)-C(43)-H(43A)	109.5
C(40)-C(43)-H(43B)	109.5
H(43A)-C(43)-H(43B)	109.5
C(40)-C(43)-H(43C)	109.5
H(43A)-C(43)-H(43C)	109.5
H(43B)-C(43)-H(43C)	109.5
C(45)-C(44)-C(57)	120.7(7)
C(45)-C(44)-C(7)	119.6(7)
C(57)-C(44)-C(7)	118.8(7)
C(44)-C(45)-C(46)	119.9(7)
C(44)-C(45)-H(45)	120.1

C(46)-C(45)-H(45)	120.1
C(45)-C(46)-C(47)	123.8(7)
C(45)-C(46)-C(18)	118.8(7)
C(47)-C(46)-C(18)	117.4(8)
C(48)-C(47)-C(46)	125.1(7)
C(48)-C(47)-C(58)	121.7(6)
C(46)-C(47)-C(58)	112.8(8)
C(49)-C(48)-C(47)	122.7(7)
C(49)-C(48)-H(48)	118.6
C(47)-C(48)-H(48)	118.6
C(48)-C(49)-C(50)	120.6(8)
C(48)-C(49)-H(49)	119.7
C(50)-C(49)-H(49)	119.7
C(59)-C(50)-C(51)	118.9(6)
C(59)-C(50)-C(49)	119.3(6)
C(51)-C(50)-C(49)	121.8(7)
C(52)-C(51)-C(28)	122.5(7)
C(52)-C(51)-C(50)	117.3(7)
C(28)-C(51)-C(50)	120.1(7)
C(51)-C(52)-C(53)	124.6(7)
C(51)-C(52)-H(52)	117.7
C(53)-C(52)-H(52)	117.7
C(54)-C(53)-C(52)	117.8(7)
C(54)-C(53)-C(39)	124.5(7)
C(52)-C(53)-C(39)	117.7(7)
C(53)-C(54)-C(55)	122.0(7)
C(53)-C(54)-C(59)	119.7(8)
C(55)-C(54)-C(59)	118.2(7)
C(56)-C(55)-C(54)	123.2(7)
C(56)-C(55)-H(55)	118.4
C(54)-C(55)-H(55)	118.4
C(55)-C(56)-C(57)	120.3(8)
C(55)-C(56)-H(56)	119.8
C(57)-C(56)-H(56)	119.8
C(58)-C(57)-C(44)	118.1(7)
C(58)-C(57)-C(56)	120.1(7)

C(44)-C(57)-C(56)	121.8(7)
C(57)-C(58)-C(47)	124.7(7)
C(57)-C(58)-C(59)	120.6(7)
C(47)-C(58)-C(59)	114.6(7)
C(50)-C(59)-C(54)	121.6(7)
C(50)-C(59)-C(58)	120.7(6)
C(54)-C(59)-C(58)	117.6(7)
O(14)-C(60)-O(13)	124.1(18)
O(14)-C(60)-C(61)	120.6(16)
O(13)-C(60)-C(61)	114.3(14)
O(15)-C(61)-C(62)	105.7(11)
O(15)-C(61)-C(60)	110.5(10)
C(62)-C(61)-C(60)	105.3(12)
O(15)-C(61)-H(61)	111.7
C(62)-C(61)-H(61)	111.7
C(60)-C(61)-H(61)	111.7
C(61)-C(62)-H(62A)	109.5
C(61)-C(62)-H(62B)	109.5
H(62A)-C(62)-H(62B)	109.5
C(61)-C(62)-H(62C)	109.5
H(62A)-C(62)-H(62C)	109.5
H(62B)-C(62)-H(62C)	109.5
C(64)-C(63)-O(15)	124.8(9)
C(64)-C(63)-C(68)	122.0(9)
O(15)-C(63)-C(68)	113.2(10)
C(63)-C(64)-C(65)	117.4(9)
C(63)-C(64)-H(64)	121.3
C(65)-C(64)-H(64)	121.3
C(66)-C(65)-C(64)	120.2(9)
C(66)-C(65)-C(72)	122.1(8)
C(64)-C(65)-C(72)	117.6(10)
C(65)-C(66)-C(67)	119.7(8)
C(65)-C(66)-C(70)	120.5(8)
C(67)-C(66)-C(70)	119.8(7)
C(68)-C(67)-C(66)	119.1(9)
C(68)-C(67)-C(71)	122.1(8)

C(66)-C(67)-C(71)	118.8(7)
C(67)-C(68)-C(63)	121.3(10)
C(67)-C(68)-H(68)	119.3
C(63)-C(68)-H(68)	119.3
C(70)#7-C(69)-C(70)	120.4(11)
C(70)#7-C(69)-H(69)	119.8
C(70)-C(69)-H(69)	119.8
C(69)-C(70)-C(73)	120.6(8)
C(69)-C(70)-C(66)	118.7(8)
C(73)-C(70)-C(66)	120.7(7)
C(67)-C(71)-H(71A)	109.5
C(67)-C(71)-H(71B)	109.5
H(71A)-C(71)-H(71B)	109.5
C(67)-C(71)-H(71C)	109.5
H(71A)-C(71)-H(71C)	109.5
H(71B)-C(71)-H(71C)	109.5
C(65)-C(72)-H(72A)	109.5
C(65)-C(72)-H(72B)	109.5
H(72A)-C(72)-H(72B)	109.5
C(65)-C(72)-H(72C)	109.5
H(72A)-C(72)-H(72C)	109.5
H(72B)-C(72)-H(72C)	109.5
C(74)-C(73)-C(78)	123.2(8)
C(74)-C(73)-C(70)	116.3(8)
C(78)-C(73)-C(70)	120.2(7)
C(73)-C(74)-C(73)#7	125.6(11)
C(73)-C(74)-C(75)	117.2(6)
C(73)#7-C(74)-C(75)	117.2(6)
C(74)-C(75)-C(76)	121.4(5)
C(74)-C(75)-C(76)#7	121.4(5)
C(76)-C(75)-C(76)#7	117.3(10)
C(80)-C(76)-C(77)	126.1(8)
C(80)-C(76)-C(75)	120.2(8)
C(77)-C(76)-C(75)	113.8(8)
C(78)-C(77)-C(76)	126.3(8)
C(78)-C(77)-H(77)	116.9

C(76)-C(77)-H(77)	116.9
C(77)-C(78)-C(73)	118.0(8)
C(77)-C(78)-H(78)	121.0
C(73)-C(78)-H(78)	121.0
C(80)-C(79)-C(80)#7	128.4(15)
C(80)-C(79)-H(79)	115.8
C(80)#7-C(79)-H(79)	115.8
C(79)-C(80)-C(76)	117.0(10)
C(79)-C(80)-C(81)	125.7(10)
C(76)-C(80)-C(81)	117.2(8)
C(82)-C(81)-C(86)	114.4(9)
C(82)-C(81)-C(80)	122.5(8)
C(86)-C(81)-C(80)	122.6(8)
C(81)-C(82)-C(83)	124.8(9)
C(81)-C(82)-C(88)	115.9(8)
C(83)-C(82)-C(88)	118.9(9)
C(84)-C(83)-C(82)	118.0(10)
C(84)-C(83)-H(83)	121.0
C(82)-C(83)-H(83)	121.0
C(83)-C(84)-O(18)	119.8(12)
C(83)-C(84)-C(85)	120.4(9)
O(18)-C(84)-C(85)	119.4(11)
C(86)-C(85)-C(84)	122.2(10)
C(86)-C(85)-H(85)	118.9
C(84)-C(85)-H(85)	118.9
C(85)-C(86)-C(81)	118.9(10)
C(85)-C(86)-C(87)	125.7(10)
C(81)-C(86)-C(87)	115.2(8)
C(86)-C(87)-H(87A)	109.5
C(86)-C(87)-H(87B)	109.5
H(87A)-C(87)-H(87B)	109.5
C(86)-C(87)-H(87C)	109.5
H(87A)-C(87)-H(87C)	109.5
H(87B)-C(87)-H(87C)	109.5
C(82)-C(88)-H(88A)	109.5
C(82)-C(88)-H(88B)	109.5

H(88A)-C(88)-H(88B)	109.5
C(82)-C(88)-H(88C)	109.5
H(88A)-C(88)-H(88C)	109.5
H(88B)-C(88)-H(88C)	109.5
C(24)-C(94)-H(94A)	109.5
C(24)-C(94)-H(94B)	109.5
H(94A)-C(94)-H(94B)	109.5
C(24)-C(94)-H(94C)	109.5
H(94A)-C(94)-H(94C)	109.5
H(94B)-C(94)-H(94C)	109.5
C(97)-C(95)-H(95A)	109.5
C(97)-C(95)-H(95B)	109.5
H(95A)-C(95)-H(95B)	109.5
C(97)-C(95)-H(95C)	109.5
H(95A)-C(95)-H(95C)	109.5
H(95B)-C(95)-H(95C)	109.5
C(97)-C(96)-O(16)	123.7(19)
C(97)-C(96)-O(17)	134.4(19)
O(16)-C(96)-O(17)	98.0(16)
C(96)-C(97)-O(18)	115.3(18)
C(96)-C(97)-C(95)	101.7(12)
O(18)-C(97)-C(95)	111.0(13)
C(96)-C(97)-H(97)	109.5
O(18)-C(97)-H(97)	109.5
C(95)-C(97)-H(97)	109.5
C(92)-N(1)-C(93)	102.3(9)
C(92)-N(1)-H(1A)	111.2
C(93)-N(1)-H(1A)	110.8
C(92)-N(1)-H(1B)	112.2
C(93)-N(1)-H(1B)	111.0
H(1A)-N(1)-H(1B)	109.1
N(1)-C(92)-H(92A)	109.5
N(1)-C(92)-H(92B)	109.5
H(92A)-C(92)-H(92B)	109.5
N(1)-C(92)-H(92C)	109.5
H(92A)-C(92)-H(92C)	109.5

H(92B)-C(92)-H(92C)	109.5
N(1)-C(93)-H(93A)	109.5
N(1)-C(93)-H(93B)	109.5
H(93A)-C(93)-H(93B)	109.5
N(1)-C(93)-H(93C)	109.5
H(93A)-C(93)-H(93C)	109.5
H(93B)-C(93)-H(93C)	109.5

Symmetry transformations used to generate equivalent atoms:

#1 x-1/2,y-1/2,z+1/2   #2 -x+1/2,y-1/2,-z+1/2

#3 -x+3/2,y-1/2,-z+1/2   #4 -x+1/2,y+1/2,-z+1/2

#5 x+1/2,y+1/2,z-1/2   #6 -x+3/2,y+1/2,-z+1/2

#7 -x,y,-z+1

Table S5. Anisotropic displacement parameters ($Å^2$x 10^3) for **Zn-PLA**. The anisotropic displacement factor exponent takes the form: $-2\pi^2[h^2a^{*2}U^{11} + ... + 2 h k a^* b^* U^{12}]$

	U^{11}	U^{22}	U^{33}	U^{23}	U^{13}	U^{12}
Zn(1)	74(1)	90(1)	92(1)	25(1)	32(1)	4(1)
Zn(2)	314(4)	398(7)	249(3)	90(4)	141(3)	153(5)
O(1)	104(6)	267(15)	89(5)	42(7)	25(5)	19(7)
O(2)	137(6)	87(7)	155(7)	19(6)	54(6)	-6(5)
O(3)	78(4)	53(5)	162(6)	29(5)	31(4)	-8(4)
O(4)	97(5)	140(9)	98(5)	22(6)	-3(4)	38(5)
O(5)	95(5)	139(8)	112(5)	-17(5)	36(4)	-21(5)
O(6)	85(5)	99(6)	79(4)	-9(4)	-4(3)	9(4)
O(7)	120(6)	117(7)	116(6)	5(5)	34(5)	-25(5)
O(8)	153(8)	74(6)	109(6)	30(5)	21(6)	12(6)
O(9)	207(9)	127(8)	85(5)	-15(5)	21(5)	-103(7)
O(10)	138(8)	181(12)	203(10)	64(10)	77(7)	47(8)
O(11)	143(8)	199(14)	171(9)	93(9)	73(7)	40(8)
O(12)	75(5)	159(9)	131(6)	65(6)	20(4)	13(5)
O(13)	83(5)	160(9)	98(5)	-1(5)	1(4)	23(6)
O(14)	119(6)	117(8)	202(9)	5(9)	-18(6)	24(7)
O(15)	120(6)	102(7)	84(4)	-15(4)	38(4)	39(5)
O(16)	166(4)	189(6)	129(3)	1(4)	77(3)	-18(4)
O(17)	166(4)	189(6)	129(3)	1(4)	77(3)	-18(4)
O(18)	166(4)	189(6)	129(3)	1(4)	77(3)	-18(4)
O(19)	204(9)	42(5)	170(7)	30(5)	84(7)	25(6)
O(20)	180(14)	470(40)	630(50)	-100(40)	160(20)	-60(20)
C(1)	146(12)	150(15)	65(7)	34(8)	0(8)	-33(10)
C(2)	70(6)	78(9)	158(10)	34(7)	77(7)	16(6)
C(3)	132(10)	112(12)	175(12)	48(10)	44(9)	-65(9)
C(4)	55(5)	81(10)	125(8)	60(8)	17(5)	8(6)
C(5)	73(6)	44(7)	81(6)	23(5)	20(5)	-2(5)
C(6)	82(6)	49(7)	72(5)	2(5)	3(5)	4(5)
C(7)	52(5)	48(7)	74(5)	26(5)	-5(4)	13(4)
C(8)	85(6)	28(6)	85(6)	29(5)	8(5)	7(5)
C(9)	72(6)	69(9)	116(7)	-8(6)	17(5)	-2(6)

C(10)	158(11)	96(11)	94(7)	6(7)	39(7)	33(9)
C(11)	142(9)	52(7)	59(5)	5(5)	7(5)	-13(6)
C(12)	148(13)	118(16)	151(14)	25(12)	67(11)	23(12)
C(13)	91(9)	210(20)	179(14)	87(13)	46(9)	44(11)
C(14)	97(10)	159(16)	216(15)	56(13)	-22(9)	15(10)
C(15)	43(6)	177(15)	77(7)	59(8)	-17(5)	-44(8)
C(16)	87(9)	142(13)	88(8)	70(8)	34(7)	50(8)
C(17)	74(7)	74(8)	76(6)	35(6)	15(5)	5(6)
C(18)	56(5)	99(9)	73(6)	49(6)	15(4)	31(6)
C(19)	87(7)	86(8)	39(4)	-3(5)	0(4)	-46(6)
C(20)	28(5)	145(11)	67(5)	20(6)	-1(4)	-26(6)
C(21)	138(10)	107(11)	91(7)	-24(7)	29(7)	4(8)
C(22)	52(5)	67(7)	95(6)	-36(6)	20(4)	-8(5)
C(23)	130(11)	202(19)	76(8)	-55(10)	48(8)	-87(13)
C(24)	127(10)	222(19)	102(9)	-19(10)	44(8)	-87(12)
C(25)	116(9)	76(10)	69(6)	7(7)	-5(6)	-45(8)
C(26)	69(5)	95(9)	93(7)	83(8)	20(5)	-1(7)
C(27)	61(5)	66(7)	65(5)	-3(5)	-26(4)	3(5)
C(28)	66(5)	20(5)	58(4)	-12(4)	1(4)	1(4)
C(29)	42(5)	97(10)	73(6)	25(6)	4(4)	-11(5)
C(30)	109(7)	74(8)	49(5)	-25(5)	2(5)	-3(7)
C(31)	124(9)	100(12)	138(10)	-12(9)	54(7)	32(8)
C(32)	126(8)	59(7)	71(5)	-11(5)	24(5)	-3(6)
C(33)	97(8)	61(8)	68(6)	19(6)	4(6)	4(7)
C(34)	43(5)	131(12)	91(6)	-17(7)	-6(5)	-13(6)
C(35)	96(9)	230(20)	176(13)	-83(14)	15(8)	-28(11)
C(36)	57(6)	81(8)	52(5)	12(5)	-3(4)	-12(5)
C(37)	81(7)	63(8)	88(6)	-23(6)	18(6)	-8(6)
C(38)	58(5)	39(6)	86(6)	-4(5)	-1(4)	-2(4)
C(39)	45(5)	38(6)	69(5)	18(4)	-13(4)	-13(4)
C(40)	65(6)	66(7)	56(5)	3(4)	-1(4)	10(5)
C(41)	45(5)	124(11)	85(6)	20(7)	17(5)	16(6)
C(42)	103(8)	108(11)	121(8)	-58(8)	24(7)	-4(7)
C(43)	88(7)	63(8)	91(6)	-20(6)	-1(5)	24(6)
C(44)	85(6)	17(5)	74(5)	15(4)	-6(5)	-6(4)
C(45)	51(5)	46(6)	55(4)	24(4)	-16(4)	-12(4)

C(46)	56(5)	43(6)	60(5)	-3(4)	-2(4)	12(4)
C(47)	57(5)	54(6)	52(4)	18(4)	5(4)	24(4)
C(48)	61(5)	66(7)	42(4)	8(4)	-20(4)	12(5)
C(49)	75(6)	35(6)	63(5)	2(4)	6(4)	1(5)
C(50)	45(4)	24(5)	56(4)	1(4)	-3(3)	-8(4)
C(51)	66(5)	20(5)	63(4)	18(4)	0(4)	10(4)
C(52)	61(5)	50(6)	54(4)	9(4)	-14(4)	3(4)
C(53)	56(5)	33(6)	68(5)	-6(4)	3(4)	2(4)
C(54)	67(5)	44(6)	45(4)	-15(4)	-3(4)	3(4)
C(55)	73(5)	42(6)	60(5)	25(4)	-31(4)	-15(5)
C(56)	74(6)	26(6)	74(5)	-18(4)	12(4)	-9(4)
C(57)	64(5)	26(5)	69(5)	16(4)	17(4)	0(4)
C(58)	48(4)	42(6)	57(4)	0(4)	1(3)	17(4)
C(59)	59(5)	43(6)	49(4)	-6(4)	-14(3)	-12(4)
C(60)	152(15)	130(15)	135(11)	-34(11)	65(11)	-37(12)
C(61)	191(14)	98(11)	78(7)	58(7)	-3(8)	45(10)
C(62)	252(19)	370(30)	66(7)	-22(11)	43(9)	150(20)
C(63)	93(8)	45(7)	91(7)	6(6)	24(6)	9(6)
C(64)	85(7)	37(6)	102(7)	16(6)	33(6)	10(5)
C(65)	114(9)	39(7)	84(6)	-12(5)	20(6)	-9(6)
C(66)	45(5)	29(5)	73(5)	13(4)	18(4)	12(4)
C(67)	107(7)	15(5)	69(5)	-19(4)	10(5)	0(5)
C(68)	105(8)	53(7)	63(5)	3(5)	11(5)	14(6)
C(69)	71(7)	15(7)	60(6)	0	5(5)	0
C(70)	53(5)	50(7)	82(5)	-2(5)	8(4)	-10(4)
C(71)	105(7)	61(7)	49(4)	-3(4)	3(4)	24(6)
C(72)	104(8)	99(10)	90(7)	-32(7)	-1(6)	0(7)
C(73)	49(4)	22(5)	63(4)	-3(4)	12(3)	-2(4)
C(74)	51(6)	35(8)	77(7)	0	1(6)	0
C(75)	39(6)	24(7)	68(7)	0	-4(5)	0
C(76)	58(5)	39(6)	50(4)	-11(4)	-2(4)	1(4)
C(77)	59(5)	47(7)	67(5)	5(5)	-2(4)	4(5)
C(78)	68(5)	39(6)	68(5)	-4(5)	4(4)	6(5)
C(80)	71(6)	36(6)	67(5)	-4(5)	18(4)	10(5)
C(81)	88(6)	35(6)	71(5)	-6(5)	13(5)	4(5)
C(82)	77(6)	50(7)	80(6)	9(5)	-3(5)	23(5)

C(83)	120(9)	61(8)	90(7)	28(6)	41(6)	-5(7)
C(84)	123(9)	47(7)	88(7)	39(6)	63(7)	27(7)
C(85)	135(10)	59(8)	120(9)	29(7)	65(8)	-10(7)
C(86)	77(6)	34(6)	98(7)	-9(5)	19(5)	-8(5)
C(87)	116(9)	92(10)	139(10)	11(8)	33(8)	-24(8)
C(88)	110(8)	149(13)	118(9)	67(9)	-38(7)	-33(8)
C(94)	207(15)	230(20)	146(11)	-28(12)	71(11)	-174(16)
C(95)	166(4)	189(6)	129(3)	1(4)	77(3)	-18(4)
C(96)	166(4)	189(6)	129(3)	1(4)	77(3)	-18(4)
C(97)	166(4)	189(6)	129(3)	1(4)	77(3)	-18(4)
N(1)	101(7)	200(13)	111(6)	-30(9)	13(6)	43(8)
C(92)	113(9)	320(30)	113(10)	-12(12)	-7(8)	65(12)
C(93)	169(13)	230(20)	147(11)	-44(15)	-16(10)	-16(16)

Table S6. Torsion angles [°] for **Zn-PLA**.

Zn(1)-O(2)-C(1)-O(1)	2(2)
Zn(1)-O(2)-C(1)-C(2)	-167.0(7)
C(4)-O(3)-C(2)-C(1)	87.4(11)
C(4)-O(3)-C(2)-C(3)	-151.6(9)
O(1)-C(1)-C(2)-O(3)	39.6(17)
O(2)-C(1)-C(2)-O(3)	-150.3(11)
O(1)-C(1)-C(2)-C(3)	-76.3(16)
O(2)-C(1)-C(2)-C(3)	93.7(15)
C(2)-O(3)-C(4)-C(5)	-26.5(13)
C(2)-O(3)-C(4)-C(9)	163.4(8)
O(3)-C(4)-C(5)-C(6)	-176.6(8)
C(9)-C(4)-C(5)-C(6)	-6.7(13)
C(4)-C(5)-C(6)-C(7)	2.3(13)
C(4)-C(5)-C(6)-C(10)	-174.0(9)
C(5)-C(6)-C(7)-C(8)	0.1(12)
C(10)-C(6)-C(7)-C(8)	176.6(8)
C(5)-C(6)-C(7)-C(44)	-178.0(8)
C(10)-C(6)-C(7)-C(44)	-1.6(12)
C(6)-C(7)-C(8)-C(9)	2.2(12)
C(44)-C(7)-C(8)-C(9)	-179.6(8)
C(6)-C(7)-C(8)-C(11)	-179.7(8)
C(44)-C(7)-C(8)-C(11)	-1.5(12)
C(7)-C(8)-C(9)-C(4)	-7.1(14)
C(11)-C(8)-C(9)-C(4)	174.7(8)
O(3)-C(4)-C(9)-C(8)	-179.8(9)
C(5)-C(4)-C(9)-C(8)	9.5(14)
Zn(2)-O(10)-C(12)-O(11)	-2(2)
Zn(2)-O(10)-C(12)-C(13)	170.8(10)
O(11)-C(12)-C(13)-O(12)	24(2)
O(10)-C(12)-C(13)-O(12)	-148.4(15)
O(11)-C(12)-C(13)-C(14)	-93(2)
O(10)-C(12)-C(13)-C(14)	94.5(18)
C(15)-O(12)-C(13)-C(12)	70.0(16)
C(15)-O(12)-C(13)-C(14)	-173.4(10)

C(13)-O(12)-C(15)-C(16)	1.2(14)
C(13)-O(12)-C(15)-C(20)	-175.8(9)
C(20)-C(15)-C(16)-C(17)	-2.1(16)
O(12)-C(15)-C(16)-C(17)	-178.8(8)
C(15)-C(16)-C(17)-C(18)	2.6(13)
C(15)-C(16)-C(17)-C(21)	176.3(10)
C(16)-C(17)-C(18)-C(19)	-1.3(12)
C(21)-C(17)-C(18)-C(19)	-174.9(9)
C(16)-C(17)-C(18)-C(46)	172.8(7)
C(21)-C(17)-C(18)-C(46)	-0.7(12)
C(17)-C(18)-C(19)-C(20)	-0.5(12)
C(46)-C(18)-C(19)-C(20)	-174.3(7)
C(17)-C(18)-C(19)-C(22)	-179.8(8)
C(46)-C(18)-C(19)-C(22)	6.4(12)
C(18)-C(19)-C(20)-C(15)	1.2(13)
C(22)-C(19)-C(20)-C(15)	-179.5(9)
C(16)-C(15)-C(20)-C(19)	0.0(15)
O(12)-C(15)-C(20)-C(19)	177.0(8)
Zn(1)#5-O(7)-C(23)-O(8)	0(2)
Zn(1)#5-O(7)-C(23)-C(24)	-174.4(9)
C(25)-O(9)-C(24)-C(23)	65.8(18)
C(25)-O(9)-C(24)-C(94)	-176.4(10)
O(8)-C(23)-C(24)-O(9)	31(2)
O(7)-C(23)-C(24)-O(9)	-154.2(13)
O(8)-C(23)-C(24)-C(94)	-80.8(17)
O(7)-C(23)-C(24)-C(94)	93.7(18)
C(24)-O(9)-C(25)-C(30)	172.7(10)
C(24)-O(9)-C(25)-C(26)	-7.6(16)
C(30)-C(25)-C(26)-C(27)	2.2(16)
O(9)-C(25)-C(26)-C(27)	-177.6(8)
C(25)-C(26)-C(27)-C(28)	-1.3(12)
C(25)-C(26)-C(27)-C(31)	-179.9(10)
C(26)-C(27)-C(28)-C(29)	1.4(11)
C(31)-C(27)-C(28)-C(29)	-179.9(8)
C(26)-C(27)-C(28)-C(51)	179.6(7)
C(31)-C(27)-C(28)-C(51)	-1.7(11)

C(27)-C(28)-C(29)-C(30)	-2.4(12)
C(51)-C(28)-C(29)-C(30)	179.4(7)
C(27)-C(28)-C(29)-C(32)	-174.9(7)
C(51)-C(28)-C(29)-C(32)	6.8(13)
C(26)-C(25)-C(30)-C(29)	-3.6(18)
O(9)-C(25)-C(30)-C(29)	176.2(9)
C(28)-C(29)-C(30)-C(25)	3.6(15)
C(32)-C(29)-C(30)-C(25)	176.1(10)
Zn(1)#4-O(5)-C(33)-O(4)	-1.2(15)
Zn(1)#4-O(5)-C(33)-C(34)	173.2(6)
C(36)-O(6)-C(34)-C(33)	78.9(11)
C(36)-O(6)-C(34)-C(35)	-161.1(10)
O(4)-C(33)-C(34)-O(6)	14.7(13)
O(5)-C(33)-C(34)-O(6)	-160.4(8)
O(4)-C(33)-C(34)-C(35)	-99.8(11)
O(5)-C(33)-C(34)-C(35)	85.1(11)
C(34)-O(6)-C(36)-C(37)	170.2(10)
C(34)-O(6)-C(36)-C(41)	-9.0(13)
C(41)-C(36)-C(37)-C(38)	1.3(16)
O(6)-C(36)-C(37)-C(38)	-177.9(9)
C(36)-C(37)-C(38)-C(39)	-5.1(15)
C(36)-C(37)-C(38)-C(42)	179.4(10)
C(37)-C(38)-C(39)-C(40)	5.3(13)
C(42)-C(38)-C(39)-C(40)	-179.3(9)
C(37)-C(38)-C(39)-C(53)	-179.4(8)
C(42)-C(38)-C(39)-C(53)	-4.1(12)
C(38)-C(39)-C(40)-C(41)	-1.8(13)
C(53)-C(39)-C(40)-C(41)	-176.9(8)
C(38)-C(39)-C(40)-C(43)	177.9(8)
C(53)-C(39)-C(40)-C(43)	2.9(12)
C(37)-C(36)-C(41)-C(40)	2.4(15)
O(6)-C(36)-C(41)-C(40)	-178.4(9)
C(39)-C(40)-C(41)-C(36)	-2.0(14)
C(43)-C(40)-C(41)-C(36)	178.2(8)
C(6)-C(7)-C(44)-C(45)	86.2(10)
C(8)-C(7)-C(44)-C(45)	-92.0(9)

C(6)-C(7)-C(44)-C(57)	-104.1(9)
C(8)-C(7)-C(44)-C(57)	77.7(10)
C(57)-C(44)-C(45)-C(46)	-0.5(13)
C(7)-C(44)-C(45)-C(46)	169.0(8)
C(44)-C(45)-C(46)-C(47)	0.9(13)
C(44)-C(45)-C(46)-C(18)	-179.0(8)
C(19)-C(18)-C(46)-C(45)	-105.1(10)
C(17)-C(18)-C(46)-C(45)	80.5(9)
C(19)-C(18)-C(46)-C(47)	74.9(10)
C(17)-C(18)-C(46)-C(47)	-99.5(9)
C(45)-C(46)-C(47)-C(48)	-174.2(8)
C(18)-C(46)-C(47)-C(48)	5.8(12)
C(45)-C(46)-C(47)-C(58)	-1.7(11)
C(18)-C(46)-C(47)-C(58)	178.2(7)
C(46)-C(47)-C(48)-C(49)	177.9(8)
C(58)-C(47)-C(48)-C(49)	6.0(13)
C(47)-C(48)-C(49)-C(50)	-3.5(12)
C(48)-C(49)-C(50)-C(59)	2.2(11)
C(48)-C(49)-C(50)-C(51)	-176.7(7)
C(29)-C(28)-C(51)-C(52)	84.7(11)
C(27)-C(28)-C(51)-C(52)	-93.5(10)
C(29)-C(28)-C(51)-C(50)	-92.4(10)
C(27)-C(28)-C(51)-C(50)	89.4(9)
C(59)-C(50)-C(51)-C(52)	1.1(11)
C(49)-C(50)-C(51)-C(52)	-180.0(7)
C(59)-C(50)-C(51)-C(28)	178.4(7)
C(49)-C(50)-C(51)-C(28)	-2.7(11)
C(28)-C(51)-C(52)-C(53)	179.4(8)
C(50)-C(51)-C(52)-C(53)	-3.4(12)
C(51)-C(52)-C(53)-C(54)	3.9(12)
C(51)-C(52)-C(53)-C(39)	-176.2(8)
C(40)-C(39)-C(53)-C(54)	81.7(11)
C(38)-C(39)-C(53)-C(54)	-93.5(9)
C(40)-C(39)-C(53)-C(52)	-98.2(9)
C(38)-C(39)-C(53)-C(52)	86.7(9)
C(52)-C(53)-C(54)-C(55)	-179.2(8)

C(39)-C(53)-C(54)-C(55)	0.9(12)
C(52)-C(53)-C(54)-C(59)	-2.0(11)
C(39)-C(53)-C(54)-C(59)	178.1(8)
C(53)-C(54)-C(55)-C(56)	176.1(8)
C(59)-C(54)-C(55)-C(56)	-1.1(12)
C(54)-C(55)-C(56)-C(57)	-0.9(13)
C(45)-C(44)-C(57)-C(58)	1.0(12)
C(7)-C(44)-C(57)-C(58)	-168.6(8)
C(45)-C(44)-C(57)-C(56)	-178.2(8)
C(7)-C(44)-C(57)-C(56)	12.2(12)
C(55)-C(56)-C(57)-C(58)	2.0(12)
C(55)-C(56)-C(57)-C(44)	-178.8(8)
C(44)-C(57)-C(58)-C(47)	-2.1(12)
C(56)-C(57)-C(58)-C(47)	177.2(7)
C(44)-C(57)-C(58)-C(59)	179.7(7)
C(56)-C(57)-C(58)-C(59)	-1.0(11)
C(48)-C(47)-C(58)-C(57)	175.1(8)
C(46)-C(47)-C(58)-C(57)	2.3(11)
C(48)-C(47)-C(58)-C(59)	-6.6(11)
C(46)-C(47)-C(58)-C(59)	-179.3(6)
C(51)-C(50)-C(59)-C(54)	0.5(11)
C(49)-C(50)-C(59)-C(54)	-178.4(7)
C(51)-C(50)-C(59)-C(58)	175.7(6)
C(49)-C(50)-C(59)-C(58)	-3.3(11)
C(53)-C(54)-C(59)-C(50)	0.0(12)
C(55)-C(54)-C(59)-C(50)	177.3(8)
C(53)-C(54)-C(59)-C(58)	-175.3(7)
C(55)-C(54)-C(59)-C(58)	2.0(10)
C(57)-C(58)-C(59)-C(50)	-176.3(8)
C(47)-C(58)-C(59)-C(50)	5.3(10)
C(57)-C(58)-C(59)-C(54)	-0.9(10)
C(47)-C(58)-C(59)-C(54)	-179.3(7)
Zn(1)-O(13)-C(60)-O(14)	7.7(18)
Zn(1)-O(13)-C(60)-C(61)	176.6(8)
C(63)-O(15)-C(61)-C(62)	-164.8(11)
C(63)-O(15)-C(61)-C(60)	81.8(12)

O(14)-C(60)-C(61)-O(15)	10.0(18)
O(13)-C(60)-C(61)-O(15)	-159.3(10)
O(14)-C(60)-C(61)-C(62)	-103.6(16)
O(13)-C(60)-C(61)-C(62)	87.1(15)
C(61)-O(15)-C(63)-C(64)	-12.7(13)
C(61)-O(15)-C(63)-C(68)	170.1(8)
O(15)-C(63)-C(64)-C(65)	179.8(9)
C(68)-C(63)-C(64)-C(65)	-3.2(14)
C(63)-C(64)-C(65)-C(66)	-1.2(14)
C(63)-C(64)-C(65)-C(72)	-177.2(9)
C(64)-C(65)-C(66)-C(67)	2.0(13)
C(72)-C(65)-C(66)-C(67)	177.9(9)
C(64)-C(65)-C(66)-C(70)	-179.6(8)
C(72)-C(65)-C(66)-C(70)	-3.7(14)
C(65)-C(66)-C(67)-C(68)	1.7(13)
C(70)-C(66)-C(67)-C(68)	-176.8(8)
C(65)-C(66)-C(67)-C(71)	-178.8(8)
C(70)-C(66)-C(67)-C(71)	2.8(12)
C(66)-C(67)-C(68)-C(63)	-6.1(14)
C(71)-C(67)-C(68)-C(63)	174.3(9)
C(64)-C(63)-C(68)-C(67)	7.1(15)
O(15)-C(63)-C(68)-C(67)	-175.6(8)
C(70)#7-C(69)-C(70)-C(73)	0.5(5)
C(70)#7-C(69)-C(70)-C(66)	178.6(8)
C(65)-C(66)-C(70)-C(69)	-95.4(9)
C(67)-C(66)-C(70)-C(69)	83.0(9)
C(65)-C(66)-C(70)-C(73)	82.7(10)
C(67)-C(66)-C(70)-C(73)	-98.8(9)
C(69)-C(70)-C(73)-C(74)	-0.9(10)
C(66)-C(70)-C(73)-C(74)	-179.0(6)
C(69)-C(70)-C(73)-C(78)	-176.0(6)
C(66)-C(70)-C(73)-C(78)	5.8(11)
C(78)-C(73)-C(74)-C(73)#7	175.4(8)
C(70)-C(73)-C(74)-C(73)#7	0.4(5)
C(78)-C(73)-C(74)-C(75)	-4.6(8)
C(70)-C(73)-C(74)-C(75)	-179.6(5)

C(73)-C(74)-C(75)-C(76)	3.1(5)
C(73)#7-C(74)-C(75)-C(76)	-176.9(5)
C(73)-C(74)-C(75)-C(76)#7	-176.9(5)
C(73)#7-C(74)-C(75)-C(76)#7	3.1(5)
C(74)-C(75)-C(76)-C(80)	179.5(6)
C(76)#7-C(75)-C(76)-C(80)	-0.5(6)
C(74)-C(75)-C(76)-C(77)	-0.9(7)
C(76)#7-C(75)-C(76)-C(77)	179.1(7)
C(80)-C(76)-C(77)-C(78)	179.4(9)
C(75)-C(76)-C(77)-C(78)	-0.1(11)
C(76)-C(77)-C(78)-C(73)	-1.2(13)
C(74)-C(73)-C(78)-C(77)	3.7(11)
C(70)-C(73)-C(78)-C(77)	178.5(7)
C(80)#7-C(79)-C(80)-C(76)	-0.4(6)
C(80)#7-C(79)-C(80)-C(81)	176.0(10)
C(77)-C(76)-C(80)-C(79)	-178.7(6)
C(75)-C(76)-C(80)-C(79)	0.9(12)
C(77)-C(76)-C(80)-C(81)	4.6(14)
C(75)-C(76)-C(80)-C(81)	-175.9(6)
C(79)-C(80)-C(81)-C(82)	-96.0(11)
C(76)-C(80)-C(81)-C(82)	80.5(12)
C(79)-C(80)-C(81)-C(86)	74.8(12)
C(76)-C(80)-C(81)-C(86)	-108.7(10)
C(86)-C(81)-C(82)-C(83)	13.1(14)
C(80)-C(81)-C(82)-C(83)	-175.4(9)
C(86)-C(81)-C(82)-C(88)	-174.8(9)
C(80)-C(81)-C(82)-C(88)	-3.4(14)
C(81)-C(82)-C(83)-C(84)	-12.9(16)
C(88)-C(82)-C(83)-C(84)	175.3(11)
C(82)-C(83)-C(84)-O(18)	-179.5(10)
C(82)-C(83)-C(84)-C(85)	7.2(16)
C(97)-O(18)-C(84)-C(83)	168.3(13)
C(97)-O(18)-C(84)-C(85)	-18.3(18)
C(83)-C(84)-C(85)-C(86)	-3.3(17)
O(18)-C(84)-C(85)-C(86)	-176.7(10)
C(84)-C(85)-C(86)-C(81)	3.9(16)

C(84)-C(85)-C(86)-C(87)	178.2(10)
C(82)-C(81)-C(86)-C(85)	-8.3(13)
C(80)-C(81)-C(86)-C(85)	-179.8(9)
C(82)-C(81)-C(86)-C(87)	176.8(9)
C(80)-C(81)-C(86)-C(87)	5.4(13)
Zn(2)#6-O(17)-C(96)-C(97)	-105.4(18)
Zn(2)#6-O(17)-C(96)-O(16)	97.3(13)
O(16)-C(96)-C(97)-O(18)	17(2)
O(17)-C(96)-C(97)-O(18)	-135.5(18)
O(16)-C(96)-C(97)-C(95)	-103.0(17)
O(17)-C(96)-C(97)-C(95)	104.3(19)
C(84)-O(18)-C(97)-C(96)	78.0(17)
C(84)-O(18)-C(97)-C(95)	-167.1(12)

Symmetry transformations used to generate equivalent atoms:

#1 x-1/2,y-1/2,z+1/2 #2 -x+1/2,y-1/2,-z+1/2

#3 -x+3/2,y-1/2,-z+1/2 #4 -x+1/2,y+1/2,-z+1/2

#5 x+1/2,y+1/2,z-1/2 #6 -x+3/2,y+1/2,-z+1/2

#7 -x,y,-z+1
